# Estimating the Economic Value of Automated Virtual Reality Cognitive Therapy for Treating Agoraphobic Avoidance in Patients With Psychosis: Findings From the gameChange Randomized Controlled Clinical Trial

**DOI:** 10.2196/39248

**Published:** 2022-11-18

**Authors:** James Altunkaya, Michael Craven, Sinéad Lambe, Ariane Beckley, Laina Rosebrock, Robert Dudley, Kate Chapman, Anthony Morrison, Eileen O'Regan, Jenna Grabey, Aislinn Bergin, Thomas Kabir, Felicity Waite, Daniel Freeman, José Leal

**Affiliations:** 1 Health Economics Research Centre Nuffield Department of Population Health University of Oxford Oxford United Kingdom; 2 National Institute for Health and Care Research MindTech Med-Tech Co-operative Nottingham United Kingdom; 3 Human Factors Research Group Faculty of Engineering University of Nottingham Nottingham United Kingdom; 4 Mental Health & Technology Theme National Institute for Health and Care Research Nottingham Biomedical Research Centre Nottingham United Kingdom; 5 Department of Psychiatry University of Oxford Oxford United Kingdom; 6 Oxford Health NHS Foundation Trust Oxford United Kingdom; 7 Cumbria, Northumberland, Tyne, and Wear NHS Foundation Trust Newcastle upon Tyne United Kingdom; 8 Avon and Wiltshire Mental Health Partnership (AWP) NHS Trust Bath United Kingdom; 9 Greater Manchester Mental Health Foundation Trust Manchester United Kingdom; 10 Division of Psychology and Mental Health University of Manchester Manchester United Kingdom; 11 Nottinghamshire Healthcare NHS Foundation Trust Nottingham United Kingdom; 12 Oxford Primary Care Clinical Trials Unit Nuffield Department of Primary Care Health Sciences University of Oxford Oxford United Kingdom; 13 Mental Health & Clinical Neurosciences School of Medicine, Institute of Mental Health University of Nottingham Nottingham United Kingdom; 14 McPin Foundation London United Kingdom

**Keywords:** gameChange, virtual reality, National Health Service, NHS, cost-effectiveness, economic evaluation, maximum price

## Abstract

**Background:**

An automated virtual reality cognitive therapy (gameChange) has demonstrated its effectiveness to treat agoraphobia in patients with psychosis, especially for high or severe anxious avoidance. Its economic value to the health care system is not yet established.

**Objective:**

In this study, we aimed to estimate the potential economic value of gameChange for the UK National Health Service (NHS) and establish the maximum cost-effective price per patient.

**Methods:**

Using data from a randomized controlled trial with 346 patients with psychosis (ISRCTN17308399), we estimated differences in health-related quality of life, health and social care costs, and wider societal costs for patients receiving virtual reality therapy in addition to treatment as usual compared with treatment as usual alone. The maximum cost-effective prices of gameChange were calculated based on UK cost-effectiveness thresholds. The sensitivity of the results to analytical assumptions was tested.

**Results:**

Patients allocated to gameChange reported higher quality-adjusted life years (0.008 QALYs, 95% CI –0.010 to 0.026) and lower NHS and social care costs (–£105, 95% CI –£1135 to £924) compared with treatment as usual (£1=US $1.28); however, these differences were not statistically significant. gameChange was estimated to be worth up to £341 per patient from an NHS and social care (NHS and personal social services) perspective or £1967 per patient from a wider societal perspective. In patients with high or severe anxious avoidance, maximum cost-effective prices rose to £877 and £3073 per patient from an NHS and personal social services perspective and societal perspective, respectively.

**Conclusions:**

gameChange is a promising, cost-effective intervention for the UK NHS and is particularly valuable for patients with high or severe anxious avoidance. This presents an opportunity to expand cost-effective psychological treatment coverage for a population with significant health needs.

**Trial Registration:**

ISRCTN Registry ISRCTN17308399; https://www.isrctn.com/ISRCTN17308399

**International Registered Report Identifier (IRRID):**

RR2-10.1136/bmjopen-2019-031606

## Introduction

### Background

There are recognized challenges for health services providing evidence-based psychological therapies such as cognitive behavioral therapy (CBT) to patients diagnosed with psychosis [1]. There is a shortage of trained therapists in these approaches, and there are also issues of adherence and competence in therapists’ delivery of current evidence-based approaches [2,3]. The latest evidence indicates that psychosis affects approximately 0.7% of the people in the United Kingdom [4].

An automated virtual reality (VR) therapy (gameChange) was therefore recently trialed to help patients diagnosed with psychosis to re-engage with everyday situations avoided because of anxiety [5,6]. A digital coach guides the patient through the 6-session program. The automated therapy is supported by peer support workers, assistant psychologists, or clinical psychologists, enabling a much wider workforce to deliver the intervention than CBT therapists alone. The gameChange intervention led to significant reductions in anxious avoidance of, and distress in, everyday situations compared with treatment as usual (TAU) alone [6]. The largest treatment benefits were seen for patients with high or severe agoraphobic avoidance, with a corresponding clinical recommendation that these patients are prioritized within any future implementation of gameChange [6].

Nevertheless, for an intervention to be implemented in resource-constrained health systems such as the UK National Health Service (NHS), evidence of clinical benefit must be supplemented by evidence supporting a new intervention’s cost-effectiveness. This requires an intervention to be assessed using a generalizable set of economic methods that can compare the relative value of interventions across different clinical areas. This determines whether a new intervention is a cost-effective investment for the health system compared with all other potential uses of its resources.

In the United Kingdom, before a new intervention’s implementation in the NHS, the intervention’s cost-effectiveness is assessed by the National Institute for Health and Care Excellence (NICE) for England and Northern Ireland, the Scottish Medicines Consortium, and Health Technology Wales. Interventions are typically considered to be cost-effective (and therefore eligible for implementation) if they are shown to improve length of life and health-related quality of life at a cost lower than £20,000 to £30,000 (£1=US $1.28) per quality-adjusted life year (QALY) gained compared with usual care [7,8].

Although several mobile, internet, and VR CBT approaches have been previously developed and shown to be effective to varying degrees [9,10], the cost-effectiveness of only a handful of these therapies has been tested [11-15]. The economic value of only 1 VR CBT intervention has previously been tested in a population with psychosis [13], a nonautomated therapy delivered by psychologists with CBT training. By contrast, gameChange has the potential to reduce demand on clinical psychologists’ time, with its automated delivery by nonspecialist staff likely to reduce pressure on treatment waiting lists and increase rates of psychological treatment.

### Objectives

Our study examined the potential economic value of providing gameChange to patients with psychosis in the UK NHS. We used randomized controlled trial data to estimate the difference in health-related quality of life and use of NHS and wider societal resources for patients who received gameChange in addition to TAU compared with TAU alone. On the basis of the QALY thresholds of £20,000 to £30,000, we estimated a maximum cost-effective price for the gameChange intervention, especially for (largely housebound) patients with substantial agoraphobic avoidance who had been shown to experience the greatest clinical benefits [6].

## Methods

### Estimating Economic Value

We used UK national cost-effectiveness thresholds of £20,000 to £30,000 per QALY [8] to estimate the maximum cost-effective price for the gameChange intervention per patient treated for the UK health system. We compared differences in QALYs, health and social care costs, and wider societal costs between patients in the treatment and control arms of the gameChange trial to estimate the maximum price the UK health care system would be willing to pay for the gameChange intervention [16,17]. As gameChange is a new intervention, its pricing structure is currently unknown. This paper therefore presents a range of scenarios where gameChange may be implemented and the maximum cost-effective price that could be paid for an individual’s treatment with gameChange, given the health benefits observed in the trial. We present maximum cost-effective prices of the intervention at the lower bound and upper bound of the UK agencies’ willingness to pay for health at £20,000 per QALY and £30,000 per QALY, respectively.

### Data and Analysis

We estimate the costs and health outcomes of patients randomized to receive gameChange plus TAU compared with TAU alone. The gameChange trial recruited patients of NHS services with self-reported difficulties going outside because of anxiety who also had a clinical diagnosis of schizophrenia spectrum disorder or an affective diagnosis with psychotic symptoms.

Between July 25, 2019, and May 7, 2021, a total of 346 trial participants with a mean age of 37.2 (SD 12.5) years across 5 trial sites were randomized to receive the gameChange intervention (n=174, 50.3%) or TAU (n=172, 49.7%). Of the 346 patients, 232 (67.1%) were male and were recruited from 3 types of psychiatric services: early intervention (n=133, 38.4%), community mental health (n=209, 60.4%), and inpatient services (n=4, 1.2%). TAU typically comprised a prescription of antipsychotic medications, regular visits from a community mental health worker, and occasional psychiatric outpatient appointments. The gameChange intervention consisted of an automated VR cognitive therapy delivered in approximately 6 sessions of 30 minutes each over 6 weeks. The therapy aims for participants to relearn safety by undertaking repeated behavioral experiments in one of six VR social situations: a café, a general practitioner (GP) waiting room, a pub, a bus, the front door of a home, and a small local shop. Participants selected virtual tasks to complete of a graded level of social difficulty, with participants progressing through tasks with different levels of difficulty during their therapy. Full demographic information of trial participants, details of the intervention, and the outcomes collected are reported in depth elsewhere [5,6].

### Health Economic Data Collection

Resource use data were collected for each participant in the trial using questionnaires at baseline, at 6 weeks (end of VR therapy for those allocated), and at 6 months after randomization.

#### Costs

##### Health and Social Service Contacts

Participants recorded the frequency of their use of health and social care services using a client service receipt inventory self-report questionnaire adapted from previous psychiatric research at their baseline, 6-week, and 6-month interviews with trial research assistants [18]. Recorded service use consisted of GP contacts; contacts with psychiatrists, therapists, or community mental health teams; hospitalizations, including accident and emergency department visits or outpatient appointments; and use of paid help from NHS or social care services. Resource use was multiplied by unit costs (Table S1 in Multimedia Appendix 1) to estimate costs at each follow-up period. Hospital admissions were converted into an appropriate health care resource group, conditional on reason for admission, and valued using 2019-20 NHS reference costs [19]. Any length of stay beyond the health care resource group trim point was costed as excess bed days reported in 2017-18 NHS reference costs [20], inflated to the 2019 price level using the NHS pay-and-price index [21]. Admissions to mental health inpatient wards were documented by the research team from medical records and costed using NHS reference costs (Table S1 in Multimedia Appendix 1).

##### Medications and Therapies

Information on participants’ psychotropic medications and therapies was obtained by the trial team from medical record data checks at each follow-up period. The cost of all psychotropic medication prescribed during follow-up was obtained by matching reported drugs and their dose to 2019 British National Formulary prices [22]. Recorded mental health therapies were costed using a national database (Table S1 in Multimedia Appendix 1) [21].

##### Criminal Justice Services and Informal Care

Participant questionnaires captured contacts with criminal justice services (police contacts, nights spent in a prison cell or prison, psychiatric assessments received while in custody, and criminal or civil court appearances) and unpaid care received from family or friends (employment status of carers, how often they received help, and hours of care received). Table S2 in Multimedia Appendix 1 reports the unit costs used to value contacts with informal care and criminal justice services.

#### Health-Related Quality of Life

Participants completed 2 health-related quality-of-life questionnaires—EQ-5D-5L and Recovering Quality of Life, 20-item version (ReQoL-20)—at baseline, 6 weeks, and 6 months. The EQ-5D-5L determines the self-reported health status of each individual across 5 domains: mobility, self-care, usual activities, pain or discomfort, and anxiety or depression. Respondents were asked to choose one of five possible levels for each domain that reflected their *own health state today*, representing (1) *no problems*, (2) *slight problems*, (3) *moderate problems*, (4) *severe problems*, or (5) *extreme problems*. Further information on the EQ-5D-5L and its test-retest reliability is provided elsewhere [23,24]. The descriptions of respondents’ health states were converted into EQ-5D utility scores [23] using the Van Hout UK crosswalk approach to the EQ-5D-3L [25]. An alternative EQ-5D-3L crosswalk [26] was investigated in sensitivity analysis. The utility scores are truncated at 1 (full health), with 0 representing death and negative values representing states worse than death (where a person would prefer to be dead than experience a given health state).

The ReQoL-20 questionnaire consists of 20 mental health questions and 1 physical health question aimed at capturing health status for mental health conditions [27]. Further information on the ReQoL-20 questionnaire and its test-retest reliability is provided elsewhere [28]. The responses to the ReQoL-20 questionnaire were converted into ReQoL-20 utility scores [29], with the same interpretation as the EQ-5D scores: *1* represents perfect health, *0* represents death, and negative values represent states worse than death.

QALYs were calculated from health state utilities using the area under the curve approach, with utilities averaged among the time points from the baseline, 6-week, and 6-month questionnaires [30]. We produced 2 sets of QALYs: one informed by EQ-5D utilities (our base case) and a second one informed by ReQoL-20 utility scores.

#### Missing Data

We followed best practice methods for addressing missing data in cost-effectiveness studies [31]. Missing data on participant characteristics at baseline were imputed using unconditional mean imputation, and we used multiple imputation by chained equations to impute missing data on EQ-5D scores, ReQoL-20 scores, and cost components at each follow-up time point. Full details of the missing data strategy are presented in Multimedia Appendix 1 (refer to the Missing Data and Complete Case Analysis section).

### Analysis

#### Overview

Our analysis took a 6-month time horizon in line with the duration of the trial, meaning that health outcomes and costs were calculated for a duration of 6 months. Costs and health outcomes were therefore not discounted, given the short time horizon of the trial. The analysis follows intention-to-treat principles and takes 2 perspectives: first, an NHS and personal social services (PSS) perspective and, second, a societal perspective incorporating NHS and PSS costs and wider costs of criminal justice contacts, informal caregiving, and private health care expenditure. NICE’s preferred base case for estimating cost-effectiveness requires an NHS and PSS perspective and uses QALYs calculated using the EQ-5D measure [8]. Costs were analyzed at a 2019 price level, representing the price level at the start of the trial. All analyses were conducted using Stata (version 17.0; StataCorp LLC).

After multiple imputation, differences in costs between the treatment and control arms at 6 weeks and 6 months were calculated using multilevel mixed effects models, adjusted for a patients’ recruitment site and psychiatric service at randomization. The model included a time×treatment interaction, where the follow-up time point was indicated as a categorical variable. Cost differences between the arms over the whole trial period were calculated from linear regression models, including adjustment for patients’ recruitment site and psychiatric service at randomization. Differences in QALYs between the arms were calculated using the same methods, while additionally adjusting for patients’ reported baseline utility scores. Estimates derived from each imputed data set were combined using Rubin’s rules [32].

#### Calculation of Maximum Cost-effective Price

We estimated the joint uncertainty around incremental total costs and QALYs (ie, the difference between gameChange plus TAU and TAU) by bootstrapping 1000 times from each of the 22 imputed data sets (creating at least 22,000 bootstraps), running the estimation model on each bootstrapped data set, and extracting the estimated treatment effects. The maximum cost-effective price for the gameChange intervention was estimated using the net monetary benefit framework at cost-effectiveness thresholds of £20,000 per QALY and £30,000 per QALY. The net monetary benefit is the product of the mean difference in QALYs and the threshold value (representing the monetary value placed on QALY health gain by the UK health system) minus the mean difference in costs.

#### Investigating the Value of Targeting Therapy

Patients with more severe anxious avoidance and distress, as defined by their baseline Oxford Agoraphobic Avoidance Scale (OAS) scores [33], were found to have the greatest improvement in anxious avoidance of, and distress in, everyday situations between the trial arms [6]. Hence, we estimated the maximum cost-effective price for the gameChange intervention when targeted to patients with high or severe anxious avoidance or distress, as defined by their baseline OAS scores.

As avoidance and distress scores were calculated separately, we investigated 4 scenarios using each baseline measure individually, and in combination, with the intention to establish the maximum cost-effective price of gameChange in those target populations who stand the most chance to benefit from the intervention.

#### Sensitivity Analyses

We examined the impact of excluding the patients recruited from mental health inpatient services (4/346, 1.2%) who had significantly higher health care costs than the wider patient population. Furthermore, we examined the impact of using an alternative EQ-5D crosswalk function—Hernández Alava et al [26]—to estimate health utilities from patient responses to the EQ-5D-5L survey, as opposed to the Van Hout crosswalk [25] used in the main analysis. Finally, we repeated our main analysis using only complete cases (ie, individuals with cost and outcome data available in all time periods, without multiple imputation for missing data).

### Ethics Approval

The authors assert that all procedures contributing to this work comply with the ethical standards of the relevant national and institutional committees on human experimentation and with the Helsinki Declaration of 1975, as revised in 2008. All procedures involving human participants or patients were approved by the NHS Research Ethics Committee (NHS South Central-Oxford B Research Ethics Committee; 19/SC/0075). The trial was registered prospectively (ISRCTN17308399), and the trial protocol has been published [5]. Written informed consent was obtained from all participants.

## Results

### Missing Data

The percentages of missing data in each trial arm for resource use, EQ-5D utilities, and ReQoL-20 utilities at each follow-up point are presented in Table S3 in Multimedia Appendix 1. The overall percentage of missing data across all items was 22%. The levels of missing data were similar between the trial arms, and no baseline characteristics were associated with the probability of data being missing. Previous lagged utility values were also not significantly associated with the probability of utility or resource use data being missing. These results suggest that it may be plausible to assume that data are missing completely at random. As a sensitivity analysis, we therefore present a complete case analysis without imputation for missing data (Tables S4 and S5; Figures S1 and S2), replicating the results of the main analysis that were derived from 22 multiply imputed data sets.

### Costs and Health-Related Quality of Life at Each Follow-up Time Point

Table 1 presents multiply imputed costs for each category of NHS and PSS costs by treatment allocation and follow-up period, as well as adjusted mean differences, whereas Table 2 presents EQ-5D utility scores by treatment allocation at each follow-up time point, and Table 3 presents ReQoL-20 utility scores by treatment allocation at each follow-up time point. Table S6 in Multimedia Appendix 1 presents available data without imputation on reported resource use by treatment allocation and follow-up period. Table S7 in Multimedia Appendix 1 presents available data for utility values, and Table S8 in Multimedia Appendix 1 presents available data for costs. Tables S9 and S10 in Multimedia Appendix 1 present available response-level data for the EQ-5D survey and ReQoL-20 survey, respectively.

**Table 1:** Period costs by treatment allocation at each follow-up time point after multiple imputation (£1=US $1.28; N=346).^a^

	Baseline to 6 weeks				6 weeks to 6 months		
	gC^b^+TAU^c^ (n=174), mean (SE), £	TAU (n=172), mean (SE), £	Adjusted mean difference (95% CI), £	gC+TAU (n=174), mean (SE), £		TAU (n=172), mean (SE), £	Adjusted mean difference (95% CI), £
Total NHS^d^ and PSS^e^ costs	860 (205)	638 (117)	–81 (–734 to 572)	1831 (435)		1564 (414)	–36 (–694 to 622)
Mental health inpatient stays	265 (157)	105 (105)	–116 (–696 to 463)	703 (388)		551 (384)	–124 (–703 to 455)
Physical health inpatient stays	91 (91)	23 (23)	74 (–137 to 285)	147 (120)		11 (9)	141 (–70 to 352)
Medication costs	51 (7)	37 (5)	7 (–31 to 44)	168 (24)		123 (14)	37 (0 to 74)
GP^f^ visits	28 (5)	34 (5)	–9 (–24 to 7)	43 (8)		44 (5)	–3 (–19 to 13)
Psychiatrist visits	166 (35)	207 (35)	–54 (–176 to 69)	260 (35)		383 (78)	–136 (–285 to 13)
Therapist visits	21 (7)	17 (5)	2 (–43 to 48)	89 (21)		121 (26)	–34 (–80 to 12)
Community mental health	186 (25)	162 (14)	16 (–63 to 96)	361 (53)		229 (22)	124 (36 to 212)
A&E^g^ visits	5 (3)	16 (7)	–11 (–28 to 6)	14 (4)		28 (10)	–14 (–33 to 4)
Outpatient care	27 (7)	36 (8)	–9 (–33 to 16)	35 (9)		51 (12)	–16 (–42 to 10)
Paid help at home	19 (10)	1 (1)	18 (–6 to 42)	10 (8)		21 (12)	–11 (–36 to 13)

^a^Cost differences between the treatment arms were obtained from multilevel mixed effects models, adjusted for treatment allocation, randomized service, and site.

^b^gC: gameChange.

^c^TAU: treatment as usual.

^d^NHS: National Health Service.

^e^PSS: personal social services.

^f^GP: general practitioner.

^g^A&E: accident and emergency.

**Table 2:** EQ-5D utility scores by treatment allocation at each follow-up time point after multiple imputation (N=346).^a^

EQ-5D data	gC^b^+TAU^c^ (n=174), mean (SE)	TAU (n=172), mean (SE)	Adjusted mean difference (95% CI)	*P* value
Baseline	0.538 (0.021)	0.545 (0.020)	N/A^d^	N/A
6 weeks	0.608 (0.021)	0.588 (0.022)	0.026 (–0.023 to 0.075)	.30
6 months	0.570 (0.023)	0.568 (0.022)	0.007 (–0.043 to 0.057)	.78

^a^Utility differences between the treatment arms were obtained from multilevel mixed effects models, adjusted for treatment allocation, baseline utility, randomized service, and site.

^b^gC: gameChange.

^c^TAU: treatment as usual.

^d^N/A: not applicable.

**Table 3:** Recovering Quality of Life, 20-item version (ReQoL-20), utility scores by treatment allocation at each follow-up time point after multiple imputation (N=346).^a^

ReQoL-20 data	gC^b^+TAU^c^ (n=174), mean (SE)	TAU (n=172), mean (SE)	Adjusted mean difference (95% CI)	*P* value
Baseline	0.733 (0.016)	0.746 (0.015)	N/A^d^	N/A
6 weeks	0.779 (0.016)	0.765 (0.017)	0.021 (–0.017 to 0.058)	.28
6 months	0.774 (0.017)	0.792 (0.014)	–0.012 (–0.051 to 0.028)	.56

^a^A time×treatment interaction was included in both models, where the follow-up time point was used as a categorical variable.

^b^gC: gameChange.

^c^TAU: treatment as usual.

^d^N/A: not applicable.

There were no significant differences in multiply imputed NHS and PSS costs between the arms at 6 weeks or at 6 months. The cost of mental health inpatient stays was the largest driver of total NHS and PSS costs across both time periods. Participants receiving gameChange plus TAU reported higher health utility values measured using both the ReQoL-20 and the EQ-5D questionnaires at 6 weeks, although this did not reach statistical significance. However, at 6 months, the EQ-5D utility score indicated little difference between the arms, and the adjusted mean difference in the ReQoL-20 utility score indicated slightly higher utility in the control arm.

### Costs and Health Outcomes Over the Whole Trial Period

Table 4 presents QALYs calculated using EQ-5D and ReQoL-20 utilities alongside NHS and PSS as well as societal costs for the whole 6-month trial period.

There were small nonsignificant improvements in incremental QALYs for the intervention arm compared with the control arm, as measured by both health-related quality-of-life instruments. Incremental QALYs calculated using EQ-5D scores indicated a larger improvement of 0.008 QALYs (95% CI –0.010 to 0.026) for those allocated to gameChange plus TAU compared with using ReQoL-20 scores, which resulted in a gain of 0.003 QALYs (95% CI –0.011 to 0.017) compared with TAU.

No significant differences in costs were detected between the trial arms. The adjusted mean difference indicated slightly lower total NHS and PSS costs for the treatment arm compared with the control arm (–£105, 95% CI –£1135 to £924). This reduction was driven by the adjusted mean difference in the cost of NHS mental health inpatient care (–£240, 95% CI –£1098 to £617), with all other NHS and PSS cost categories indicating slightly increased mean incremental costs for the treatment arm.

From a societal perspective, cost differences between the trial arms were much larger, with an adjusted cost reduction of –£1731 (95% CI –£3886 to £424) in the intervention arm. This was driven by substantially lower costs of informal care for the treatment arm compared with the control arm.

**Table 4:** Quality-adjusted life years (QALYs) and health care and societal costs at 6 months after multiple imputation (£1=US $1.28).a

	gC^b^+TAU^c^, mean (SE)	TAU only, mean (SE)	Adjusted difference between arms, mean (95% CI)
QALYs, EQ-5D	0.293 (0.010)	0.288 (0.009)	0.008 (–0.010 to 0.026)
QALYs, ReQoL-20^d^	0.387 (0.007)	0.388 (0.006)	0.003 (–0.011 to 0.017)
Cost of mental health admissions (NHS^e^), £	969 (517)	657 (484)	–240 (–1098 to 617)
Medication costs (NHS), £	220 (30)	160 (17)	44 (–20 to 107)
General health care costs (NHS), £	1476 (237)	1356 (117)	84 (–435 to 603)
Paid help costs (NHS and PSS^f^), £	31 (15)	22 (12)	7 (–30 to 45)
Total NHS and PSS costs, £	2695 (619)	2194 (515)	–105 (–1135 to 924)
Criminal justice costs, £	42 (20)	2 (2)	38 (–0 to 77)
Unpaid caregiving (societal costs), £	2839 (400)	4403 (860)	–1576 (–3432 to 280)
Total private health care costs, £	58 (15)	135 (30)	–88 (–149 to –26)
Total societal costs, £	5634 (763)	6733 (993)	–1731 (–3886 to 424)

^a^Cost differences between the treatment arms were obtained from a linear regression model, adjusted for treatment allocation, randomized service, and site. Differences in quality-adjusted life years between the treatment arms were obtained from a linear regression model, adjusted for treatment allocation, baseline utility, randomized service, and site.

^b^gC: gameChange.

^c^TAU: treatment as usual.

^d^ReQoL-20: Recovering Quality of Life, 20-item version.

^e^NHS: National Health Service.

^f^PSS: personal social services.

### Calculation of Maximum Cost-effective Price

Table 5 reports the maximum cost-effective price for the gameChange intervention for the whole trial population. Taking an NHS and PSS perspective and using QALYs calculated using the EQ-5D, the gameChange intervention could cost up to £341 per patient at the upper NICE willingness-to-pay threshold of £30,000 per QALY. Using ReQoL-20 utilities to calculate QALYs, the gameChange intervention could cost up to £193 per patient from the NHS and PSS perspective at the same £30,000 per QALY cost-effectiveness threshold. At the lower national cost-effectiveness threshold of £20,000 per QALY, the maximum value of gameChange was £262 per patient calculated using EQ-5D QALYs or £164 per patient calculated using ReQoL-20 QALYs.

When considering the intervention’s impact on wider societal costs beyond the NHS and social care system, the maximum cost-effective price of gameChange was greater. At the upper cost-effectiveness threshold of £30,000 per QALY, gameChange could cost up to £1967 per patient using QALYs calculated using EQ-5D utilities or up to £1819 per patient using QALYs calculated using ReQoL-20 utilities. Using a threshold of £20,000 per QALY, the maximum cost was £1888 per patient using EQ-5D QALYs or £1790 per patient using ReQoL-20 QALYs.

**Figure 1:**
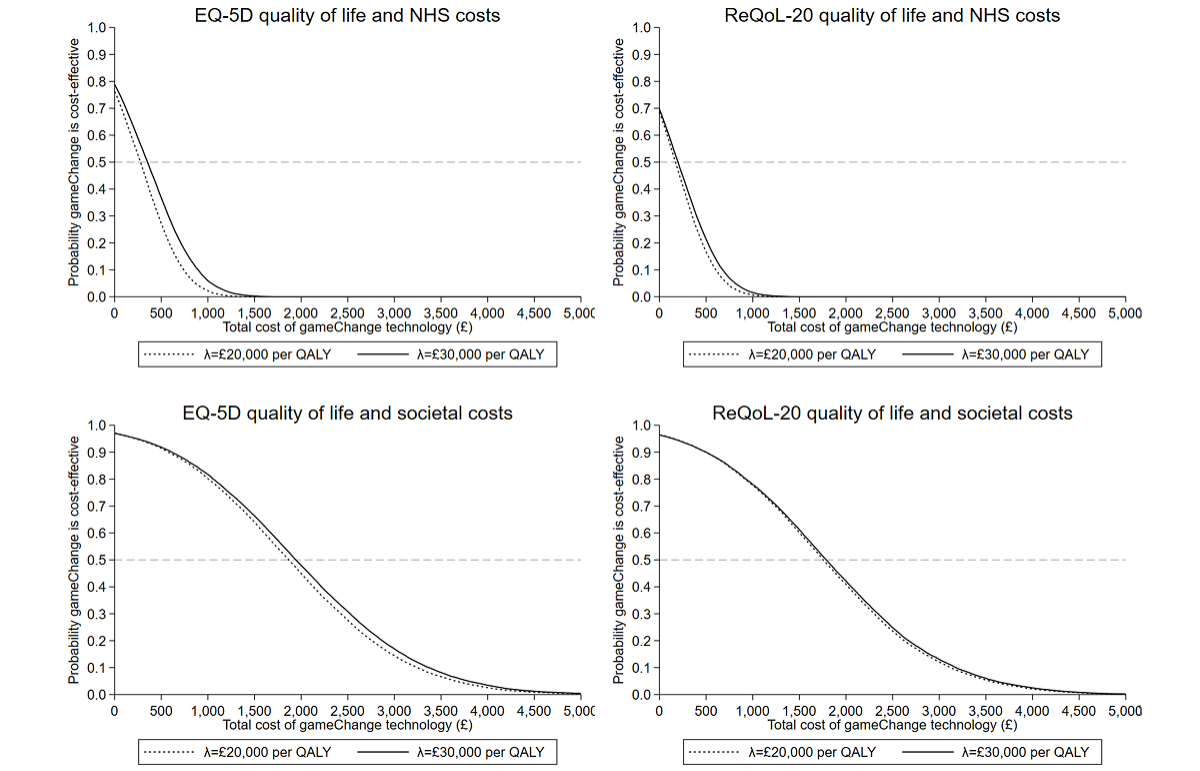
Uncertainty surrounding the maximum cost-effective price for the gameChange intervention after multiple imputation. Lines represent the maximum cost-effective price of gameChange for the UK National Health Service (NHS) at the National Institute for Health and Care Excellence cost-effectiveness threshold (between £20,000 and £30,000 per quality-adjusted life year [QALY]; £1=US $1.28). The dotted line represents the maximum cost-effective price of the gameChange intervention at the lower bound of the cost-effectiveness threshold (λ=£20,000 per QALY), whereas the solid line represents the maximum cost-effective price of the gameChange intervention at the upper bound of the cost-effectiveness threshold (λ=£30,000 per QALY). ReQoL-20: Recovering Quality of Life, 20-item version.

The probability that gameChange is cost-effective at a range of prices is presented in Figure 1, whereas uncertainty surrounding the calculations of mean costs and effects is presented in Figure S3 in Multimedia Appendix 1.

**Table 5:** Maximum cost-effective prices of gameChange using EQ-5D and ReQoL-20 utilities after multiple imputation (£1=US $1.28).^a^

Costing perspective and utilities used	Maximum cost-effective price threshold: £20,000 per QALY^b^	Maximum cost-effective price threshold: £30,000 per QALY
Total NHS^c^ and PSS^d^ costs (per NICE^e^ guidance), £, using EQ-5D utilities (per NICE guidance)	262	341
Total NHS and PSS costs (per NICE guidance), £, using ReQoL-20 utilities	164	193
Total societal costs, £, using EQ-5D utilities (per NICE guidance)	1888	1967
Total societal costs, £, using ReQoL-20 utilities	1790	1819

^a^The maximum cost-effective price of the gameChange intervention is estimated at the lower bound and upper bound of UK willingness to pay for health interventions at £20,000 per quality-adjusted life year and £30,000 per quality-adjusted life year, respectively. This represents the maximum price that can be charged for a patient’s gameChange treatment that remains cost-effective at the lower and upper bounds of the cost-effectiveness threshold.

^b^QALY: quality-adjusted life year.

^c^NHS: National Health Service.

^d^PSS: personal social services.

^e^NICE: National Institute for Health and Care Excellence.

### Investigating the Value of Targeting Therapy

Tables 6 and 7 show that more than half of the trial population were identified as having high or severe anxious avoidance (189/346, 54.6%) or distress (223/346, 64.5%) at baseline, measured using the OAS. Targeting gameChange to this patient group substantially increased the intervention’s economic value.

The maximum cost-effective price of gameChange was highest for patients with high or severe agoraphobic avoidance. At a cost-effectiveness threshold of £30,000 per QALY, gameChange could cost up to £877 per patient from an NHS and PSS perspective using EQ-5D utilities or £670 per patient using ReQoL-20 utilities. At this threshold, from a societal perspective, gameChange could cost up to £3073 per patient using EQ-5D QALYs or £2866 per patient using ReQoL-20 QALYs. The probability that gameChange is cost-effective at a range of prices is shown for each stratified scenario in Figure S4 in Multimedia Appendix 1.

**Table 6:** Maximum cost-effective price of gameChange (gC) in subgroups stratified by Oxford Agoraphobic Avoidance Scale scores after multiple imputation using the EQ-5D quality-of-life measure (£1=US $1.28; N=346).^a^

			gC+TAU^b^, n (%)			TAU, n (%)		Incremental QALY^c^ (95% CI)		Incremental cost (95% CI), £		Maximum cost-effective price, £		
												λ=20,000 per QALY		λ=30,000 per QALY
**NHS^d^ and PSS^e^ perspective**														
	Overall sample	174 (50.3)			172 (49.7)		0.008 (–0.010 to 0.026)		–105 (–1135 to 924)		262		341	
	High or severe avoidance	90 (51.7)			99 (57.6)		0.021 (–0.004 to 0.046)		–235 (–1986 to 1515)		663		877	
	High or severe distress	106 (60.9)			117 (68)		0.010 (–0.015 to 0.035)		–178 (–1683 to 1327)		374		472	
	High or severe avoidance or distress	124 (71.3)			126 (73.3)		0.014 (–0.008 to 0.037)		–98 (–1450 to 1255)		387		532	
	High or severe avoidance and distress	72 (41.4)			90 (52.3)		0.016 (–0.012 to 0.044)		–365 (–2399 to 1670)		684		844	
**Societal perspective**														
	Overall sample			174 (50.3)		172 (49.7)		0.008 (–0.010 to 0.026)		–1731 (–3886 to 424)		1888		1967
	High or severe avoidance			90 (51.7)		99 (57.6)		0.021 (–0.004 to 0.046)		–2431 (–6005 to 1142)		2859		3073
	High or severe distress			106 (60.9)		117 (68)		0.010 (–0.015 to 0.035)		–2101 (–5247 to 1045)		2297		2395
	High or severe avoidance or distress			124 (71.3)		126 (73.3)		0.014 (–0.008 to 0.037)		–2137 (–5014 to 741)		2426		2571
	High or severe avoidance and distress			72 (41.4)		90 (52.3)		0.016 (–0.012 to 0.044)		–2314 (–6398 to 1769)		2634		2794

^a^The maximum cost-effective price of the gameChange intervention is estimated at the lower (£20,000 per quality-adjusted life year) and upper (£30,000 per quality-adjusted life year) bounds of the UK cost-effectiveness threshold (λ), representing the willingness to pay for health interventions. The maximum cost-effective price represents the maximum price that can be charged for a patient’s virtual reality therapy that remains cost-effective at the lower and upper bounds of the cost-effectiveness threshold.

^b^TAU: treatment as usual.

^c^QALY: quality-adjusted life year.

^d^NHS: National Health Service.

^e^PSS: personal social services.

**Table 7:** Maximum cost-effective price of gameChange (gC) in subgroups stratified by Oxford Agoraphobic Avoidance Scale scores after multiple imputation using the Recovering Quality of Life, 20-item version, measure (£1=US $1.28; N=346).a

		gC+TAU^b^, n (%)			TAU, n (%)			Incremental QALY^c^ (95% CI)			Incremental cost (95% CI), £			Maximum cost-effective price, £				
														λ=20,000 per QALY			λ=30,000 per QALY	
**NHS^d^ and PSS^e^ perspective**																		
	Overall sample	174 (50.3)		172 (49.7)			0.003 (–0.011 to 0.017)			–105 (–1135 to 924)			164			193		
	High or severe avoidance	90 (51.7)		99 (57.6)			0.014 (–0.007 to 0.036)			–235 (–1986 to 1515)			525			670		
	High or severe distress	106 (60.9)		117 (68)			0.003 (–0.016 to 0.022)			–178 (–1683 to 1327)			240			271		
	High or severe avoidance or distress	124 (71.3)		126 (73.3)			0.008 (–0.010 to 0.026)			–98 (–1450 to 1255)			257			336		
	High or severe avoidance and distress	72 (41.4)		90 (52.3)			0.009 (–0.014 to 0.031)			–365 (–2399 to 1670)			535			620		
**Societal perspective**																		
	Overall sample		174 (50.3)			172 (49.7)			0.003 (–0.011 to 0.017)			–1731 (–3886 to 424)			1790			1819
	High or severe avoidance		90 (51.7)			99 (57.6)			0.014 (–0.007 to 0.036)			–2431 (–6005 to 1142)			2721			2866
	High or severe distress		106 (60.9)			117 (68)			0.003 (–0.016 to 0.022)			–2101 (–5247 to 1045)			2163			2194
	High or severe avoidance or distress		124 (71.3)			126 (73.3)			0.008 (–0.010 to 0.026)			–2137 (–5014 to 741)			2296			2375
	High or severe avoidance and distress		72 (41.4)			90 (52.3)			0.009 (–0.014 to 0.031)			–2314 (–6398 to 1769)			2485			2570

^a^The maximum cost-effective price of the gameChange intervention is estimated at the lower (£20,000 per quality-adjusted life year) and upper (£30,000 per quality-adjusted life year) bounds of the UK cost-effectiveness threshold (λ), representing the willingness to pay for health interventions. The maximum cost-effective price represents the maximum price that can be charged for a patient’s virtual reality therapy that remains cost-effective at the lower and upper bounds of the cost-effectiveness threshold.

^b^TAU: treatment as usual.

^c^QALY: quality-adjusted life year.

^d^NHS: National Health Service.

^e^PSS: personal social services.

### Sensitivity Analyses

The results of a sensitivity analysis excluding the participants randomized from inpatient services (4/346, 1.2%) from the analysis sample are presented in Tables S11 to S13 in Multimedia Appendix 1. Table S11 in Multimedia Appendix 1 shows that these patients have notably higher average costs. Excluding these participants from the study sample had a substantial impact, reducing the maximum cost-effective price that could be charged for gameChange across all scenarios. Table S13 and Figure S5 in Multimedia Appendix 1 show, from an NHS perspective, that gameChange was cost-effective only when targeted to those with a high or severe OAS avoidance score. From a societal perspective, gameChange was nevertheless cost-effective for all patient groups.

A separate sensitivity analysis changed the EQ-5D mapping used to estimate EQ-5D utilities, which had a small impact on the results (Table S14 in Multimedia Appendix 1 [26]; Figure S6 in Multimedia Appendix 1 [26]), slightly reducing the maximum cost-effective price that could be charged for gameChange across all scenarios considered.

Tables S4 and S5 in Multimedia Appendix 1 and Figures S1 and S2 in Multimedia Appendix 1 present results from a complete case analysis, without imputation for missing data. The results from the complete case analysis indicate slightly larger QALY differences between the arms in favor of gameChange compared with the multiply imputed analysis; however, there were also slightly lower reductions in costs. Nevertheless, the maximum cost-effective prices of gameChange are similar to the main findings from the multiply imputed analysis. The complete case analysis showed slightly less differentiation between the maximum cost-effective prices for the whole sample and the maximum cost-effective prices for patients with high or severe OAS avoidance or distress scores.

## Discussion

### Principal Findings

The gameChange intervention is likely to be of economic value to the health system, particularly when the intervention is targeted to those with high or severe anxious avoidance. This is the first study to assess the economic value of an automated VR therapy and demonstrates its potential value to a resource-constrained health system. This corroborates the findings of the single prior published randomized study that established the short-term economic value of VR CBT in a population with psychosis in the Netherlands [13]. The prior research investigated use of a nonautomated therapy that nevertheless required substantially greater therapist involvement over 16 supervised sessions, each lasting for 1 hour, compared with the automated therapy trialed in gameChange, where participants undertook a maximum of 6 VR sessions, each lasting for 30 minutes. Future research is required to establish the long-term value of both therapies within each health system because both trials only investigated quality-of-life and cost impacts of their VR intervention over a total follow-up of 6 months. It is critical to understand whether patients in receipt of VR therapies may also have improved quality of life or reduced health care costs beyond the time period of either trial, which would increase the long-term value of these interventions to the health system.

Over the 6-month trial period, the gameChange intervention is of particular value when considering a societal perspective, accounting for wider benefits beyond the health and social care system. A societal perspective is highly relevant to this patient population, who can struggle with everyday social functioning, which affects their ability to work, buy groceries, or speak with those outside their direct network. This patient group often relies on trusted friends and family to fulfill their needs, sometimes requiring significant support, which in turn affects the ability of their unpaid carers to fully contribute to society. This justifies consideration of a societal perspective when establishing the potential economic value of the gameChange intervention.

This study is based on the largest randomized trial of VR therapy to date, which collected data on many economic aspects relevant to the mental health condition studied. Our study was able to collect high-quality economic data from validated survey instruments that provide a robust assessment of changes in quality-of-life and patient costs over the 6-month trial follow-up. In particular, the diligence of the gameChange trial’s research assistant interviewers helped to minimize reporting bias in participant self-reported surveys of health and social care use, and information on medication use and mental health inpatient stays was directly collected from medical records and reviewed alongside clinical colleagues to ensure the accuracy of our cost data.

In our analysis, we calculate the potential economic value of gameChange, given its impact on health-related quality of life and in offsetting costs of health and social care use, as well as wider societal costs. Therefore, the maximum cost-effective prices we present represent the maximum potential cost per patient to fully implement the gameChange intervention that would remain a cost-effective use of health care resources. Total implementation costs will include elements such as the staff time required to administer therapy, hardware costs to purchase VR devices, and software costs of obtaining user licenses to access gameChange. These implementation costs will vary, depending on how a local provider chooses to procure and deliver the gameChange intervention. As such, local providers are best able to determine whether their expected implementation costs are below the maximum cost-effective price we project for their intended population. One model of delivery used in the gameChange trial involved an NHS band 4 staff member delivering the VR therapy predominantly at participants’ homes. We estimated that using this implementation model to deliver the entire trial caseload would cost approximately £184 per patient. Nevertheless, we anticipate that, with the decreasing cost and complexity of VR headsets, many patients could be provided a device for a period of time at home without requiring a staff member to be present at each therapy session, with patients instead supported by regular check-ins with mental health staff at lower cost.

Regarding hardware costs, the VR headsets used in the gameChange trial (HTC Vive Pro) have since been considerably superseded, with the hardware cost of VR headsets continuing to rapidly decline. The cost of a VR headset in the United Kingdom now stands at <£400, with a single device potentially being used to treat multiple patients over its life span and *per patient* prices therefore depending on expected local throughput. Software license costs to grant use of gameChange are currently locally determined, with costs subject to individual negotiation with the manufacturer. The maximum cost-effective prices we show in this analysis therefore allow individual health care providers to calculate whether the delivery model they intend to roll out for their local population at current local costs would represent a cost-effective use of health care resources in line with national UK cost-effectiveness guidelines. We believe that this approach maximizes transparency for health care providers looking to understand the value of implementing gameChange in their local population.

### Limitations

Our study includes a number of limitations. First, data on trial participants’ employment status could not be used because this data collection ceased in March 2020 at the start of the COVID-19 pandemic, which prevented us from capturing any further societal benefits of the intervention. Second, we could not control for the uneven impact of the COVID-19 pandemic on our trial outcomes because of the small numbers of participants who had completed 6-month follow-up before the pandemic. We assume that the pandemic would increase background levels of avoidance among all participants, while also affecting routine service delivery; for example, fewer GP appointments and mental health admissions being expected particularly during the first COVID-19 wave when public anxiety regarding COVID-19 and the potential infection risk from their use of health services was running high. Third, the results of a sensitivity analysis excluding trial participants randomized from psychiatric inpatient services indicate that it remains important to examine the value of gameChange for patients across different psychiatric services in future research. The small number of participants randomized from inpatient services was a major driver of the potential economic value of gameChange, with maximum cost-effective prices per patient being reduced when excluding these participants from the target population. This demonstrates the importance of adjusting for psychiatric service at randomization in the main analysis because substantially different cost and QALY profiles for participants randomized from psychiatric inpatient services likely caused skew to the mean costs and QALYs across all services. Qualitative investigation of gameChange’s implementation in inpatient services is currently underway [34]; however, the small number of participants randomized from psychiatric inpatient services limited further analytical investigation from an economic standpoint beyond the sensitivity analysis presented.

This study therefore gives health policy makers the health economic information they require to consider whether brief automated VR interventions such as gameChange should be implemented in the UK NHS. Although gameChange is shown to be economically valuable to society, its greatest value is likely to be in its ability to be implemented widely. Cost-effective interventions such as CBT and family therapy are already recommended for use in the gameChange trial population; however, only 4% (14/346) of the gameChange trial participants reported having received ≥8 sessions of either therapy at time of randomization into the trial. Because of the lack of requirement for specialist therapists, gameChange is advantaged in its potential to be cost-effective and implementable within the UK NHS, particularly when delivered by a wider supply of trained band 4 staff, which was a proven delivery method used in the gameChange trial. The gameChange intervention thus has the potential to reduce some resource pressures on the limited supply of clinical psychologists and CBT therapists, which makes gameChange potentially highly valuable to the NHS, expanding the pool of cost-effective therapies available for patients diagnosed with psychosis.

## Appendix

Multimedia Appendix 1 Supplementary appendix.

## Abbreviations

CBT: cognitive behavioral therapy
GP: general practitioner
NHS: National Health Service
NICE: National Institute for Health and Care Excellence
OAS: Oxford Agoraphobic Avoidance Scale
PSS: personal social services
QALY: quality-adjusted life year
ReQoL-20: Recovering Quality of Life, 20-item version
TAU: treatment as usual
VR: virtual reality

## Data Availability

Deidentified participant data will be available in anonymized form from the team on reasonable request (including a study outline), subject to a review and contract with Oxford Health NHS Foundation Trust, after the publication of the results.
