# Supplementary material for: Estimating the Economic Value of Automated Virtual Reality Cognitive Therapy for Treating Agoraphobic Avoidance in Patients With Psychosis: Findings From the gameChange Randomized Controlled Clinical Trial
Source: J Med Internet Res. 2022 Nov 18;24(11):e39248. doi: 10.2196/39248 (PMC9719058; doi:10.2196/39248)
Supplement: Multimedia Appendix 1 [file jmir_v24i11e39248_app1.docx]

Supplementary Appendix

Contents:

1. Unit costs
2. Missing data strategy and complete case analysis
3. Available data
4. Multiply imputed analysis
5. Sensitivity analysis 1 & 2
6. Unit costs

Table S1.Unit costs (UK 2019-20 £) of healthcare services

| **Resource use type** | **Unit cost (£)** | **Source/details** |
| --- | --- | --- |
| GP – surgery* | 39 | Cost GP patient contact consultation: PSSRU 2019-20 (chapter 10, page 124). Average consultation length of 9 minutes (Lancet. 2016;387(10035):2323-30) |
| GP – home* | 90 | Cost GP patient contact consultation (including qualification costs and direct care staff costs): PSSRU 2019-20 (chapter 10, page 124). Average consultation length of 9.22 minutes (Lancet. 2016;387(10035):2323-30); assume average 12 minutes travel time for home visits: PSSRU 2009. |
| GP nurse – surgery* | 10.85 | Cost GP nurse patient contact consultation: PSSRU 2019-20 (chapter 10, page 124). Average consultation length of 15.5 minutes: 2006/7 UK general practice workload survey |
| GP nurse– home* | 19.25 | Cost GP nurse patient contact consultation: PSSRU 2019-20 (chapter 10, page 124). Average consultation length of 15.5 minutes: 2006/7 UK general practice workload survey ; assume average 12 minutes travel time for home visits: PSSRU 2009. |
| Mental health inpatient stay (per day) | 430.63 | Weighted average of costs per occupied bed day across NHS psychosis mental health costing clusters (Clusters 10-17) reported in 2018/19 NHS reference costs, inflated to a 2019 price level using PSSRU 2020 NHSII pay and prices inflator. |
| A&E attendance | 182.28 | NHS Reference Cost schedule 2019-20, tab AE, weighted average of all attendances to a type 1 A&E unit with the exception of dental care and dead on arrival attendances. |
| Using ambulance service to attend A&E unit | 292.09 | NHS Reference Cost schedule 2019-20, tab AMB, currency code ASS02 (see and treat and convey) |
| Hospital visit to psychiatrist/psychiatrist outside hospital | 344.01 | NHS Reference Cost schedule 2019-20, tab total outpatient attendances, service code 710 (Adult mental illness), face to face attendance (WF01A and WF01B) |
| Day hospital | 168.19 | Cost day care facilities – weighted average of mental health attendances PSSRU 2010 (Chapter 7, p.119) (Cost per day). Inflated to 2019/20 cost using PSSRU 2020 inflation indices for HCS Staff (p.163) |
| Day care centre | 38 | Local authority day care for individuals requiring mental health support aged 18-64: PSSRU 2019 |
| Outpatient visit to another doctor | 153.43 | NHS Reference Cost schedule 2019-20, tab total outpatient attendances, weighted average of all service codes (except Adult mental illness) |
| Occupational therapist | 42 | 1h of NHS AfC Band 5/6 time – cost per working hour, PSSRU 2020, p.119 |
| Community psychiatric nurse | 42 | 1h of NHS AfC Band 5/6 time – cost per working hour, PSSRU 2020, p.119 |
| Counsellor/psychologist/therapist | 58 | 1h of NHS AfC Band 7 time – cost per working hour, PSSRU 2020 p.119 |
| Community mental health team coordinator | 48 | 1h of NHS AfC Band 6 time – cost per working hour, PSSRU 2020, p.119 |
| Vocational rehabilitation/recovery worker | 31 | 1h of NHS AfC Band 4 time – cost per working hour, PSSRU 2020, p.119 |
| Peer support worker | 25 | Cost support and outreach worker PSSRU 2019-20 (Chapter 11, p.144) cost per hour |
| Social worker | 51 | PSSRU 2019: social worker (adult services) cost per hour including qualifications |
| Home treatment team/crisis team | 33.41 | PSSRU 2010: Crisis resolution teams for adults with mental health problems (Chapter 12, p.185). Cost per hour, inflated to 2019/20 price level using HCS staff inflation (PSSRU 2020, p.163) |
| Non-medical prescribers | 42.62 | Cost Nurse advanced (includes lead specialist, clinical nurse specialist, senior specialist) PSSRU 2010 (Chapter 10, p.165). Cost per hour, inflated to 2019/20 price level using HCS staff inflation (PSSRU 2020,p.163) |
| Self-help or support group | 19 | Unit cost of group-based therapy led by band 5 mental health nurse. Cost of 1h session including qualifications (PSSRU 2020, p.42) |
| Paid home help/home care worker | 23 | PSSRU 2019: Home care worker cost per hour. Individual service users of domiciliary care received 7.1 hours per week (in 2014/15). |
| Complementary therapist (e.g. massage, hypnotherapy) | 48 | 1h of NHS AfC Band 6 time – cost per working hour, PSSRU 2020, p.119 |
| Alternative therapist (e.g. acupuncture, aromatherapy, Ayuvedic medicine) | 48 | 1h of NHS AfC Band 6 time – cost per working hour, PSSRU 2020, p.119 |
| **Therapies** |  |  |
| 1:1 therapies delivered by a clinical psychologist (CBT, Family Therapies, EMDR) | 106 | PSSRU 2020, p.46: Psychological therapy. Assumption of uniform cost for 1x NHS AfC Band 7 across each different therapy consistent with evidence reported in NICE CG178 and PLOS ONE 15(4): e0232245. |
| Group therapies delivered by a clinical psychologist | 23.56 | 2x 1h NHS AfC Band 7 therapists (PSSRU 2020) for average group size of 9 participants, staffing ratios as reported in NICE CG90 |
| Art or Music Therapies | 89 | 1h Band 6 NHS AfC, Cost per working hour, PSSRU 2020, p.129 |
| Behavioural Activation | 63 | 1h Band 5 NHS AfC, Cost per hour of patient-related work, PSSRU 2020, p.129, staff band reported in Health Technol Assess 2017;21(46) |
| Exercise Therapy | 11.35 | Health Technol Assess 2007;11(10), p.71, inflated to 2019 price level using the ONS composite price index |
| *including direct care staff costs and including qualification costs  NHS AfC: NHS Agenda for change pay scale – costs in 2019 price year.  PSSRU: Personal Social Services Research Unit Costs of Health and Social Care – publications for all years available at <https://www.pssru.ac.uk/project-pages/unit-costs/>  UK Office for National Statistics (ONS) composite price index available at : <https://www.ons.gov.uk/economy/inflationandpriceindices/datasets/consumerpriceinflation> | | |

**Table S2.Unit costs (UK 2019-20 £) of societal services**

| **Resource use type** | **Unit cost (£)** | **Source/details** |
| --- | --- | --- |
| Informal caregiving (employed carer) | 17.27 | Cost per hour of care received. Opportunity cost of carer’s time costed at the mean hourly wage across all UK employees in 2019. Reported in ONS: Earnings and hours worked, occupation by four-digit SOC: ASHE Table 14 |
| Informal caregiving (unemployed carer) | 8.21 | Cost per hour of care received. Opportunity cost of carer’s time costed at the minimum hourly wage in 2019. Reported in ONS: Earnings and hours worked, occupation by four-digit SOC: ASHE Table 14 |
| Contact with Police (not overnight stay) | 328 | Cost of police attendance for those who were not arrested or put under a Section 136, reported in BJ Psych, 210(2), p.157-164. Inflated to 2019 price level using the ONS composite price index |
| Nights spent in a police cell or prison | 610 | Cost of time in custody for those arrested under section 136, per custody occurrence (12h), reported in BJ Psych, 210(2), p.157-164. Inflated to 2019 price level using the ONS composite price index |
| Psychiatric assessments whilst in custody | 1300 | Cost of mental health act assessment per custody occurrence, reported in BJ Psych, 210(2), p.157-164. Inflated to 2019 price level using the ONS composite price index |
| Criminal or civil court appearances | 1471 | Total cost of mental illness to criminal justice services per case of crime committed by those with severe mental illness, reported in Lancet Public Health, 5(2), p.e99-e106. Inflated to 2019 price level using the ONS composite price index |

UK Office for National Statistics (ONS) employee earnings and hours worked available at : https://www.ons.gov.uk/employmentandlabourmarket/peopleinwork/earningsandworkinghours/datasets/occupation4digitsoc2010ashetable14

UK Office for National Statistics (ONS) composite price index available at : <https://www.ons.gov.uk/economy/inflationandpriceindices/datasets/consumerpriceinflation>

2. Missing data and complete case analysis

*Methods*

First, missing data on patient characteristics, EQ-5D, ReQoL and costs at baseline were imputed using unconditional mean imputation. Patterns of missing data in and their similarity between trial arms were then examined, in particular whether there was evidence that missing data was conditional on baseline participant characteristics (age, sex, randomised site or service, and Oxford Agoraphobic Avoidance Scale score) or on lagged outcomes (EQ-5D and ReQoL utilities). The association between probabilities of data being missing was estimated using logistic random-effects regressions. Data on psychotropic medication, mental health inpatient hospitalisations and mental health therapies was fully complete, as trial research co-ordinators completing these fields had access to all necessary patient records.

We used multiple imputation by chained equations to impute missing data on EQ-5D-5L and ReQoL utility scores, as well as cost components at each follow-up time point. Missing values were imputed as a function of age, sex, randomised site and service, Oxford Agoraphobic Avoidance scale score, baseline quality of life scores and utilities, baseline costs (by category and payer – NHS or private), and known complete data mental health therapies, inpatient stays and medication costs from the trial co-ordinators’ medical record check. The imputation model was run separately by randomised treatment. We used predictive mean matching to create 22 imputed datasets based on data from each patient’s 10 nearest neighbours. Estimates derived from each imputed dataset were combined using Rubin’s rule to estimate means with their standard error for each outcome.

**Table S3: Missing data on self-reported resource use, EQ-5D and ReQoL utilities by treatment allocation in each follow-up period**

|  | *Resource use data* | | *EQ-5D data* | | *ReQoL data* | |
| --- | --- | --- | --- | --- | --- | --- |
|  | **VR + TAU (n=174)** | **TAU (n=172)** | **VR + TAU (n=174)** | **TAU (n=172)** | **VR + TAU (n=174)** | **TAU (n=172)** |
| Baseline | 164 (94%) | 167 (97%) | 172 (99%) | 170 (99%) | 162 (93%) | 158 (92%) |
| 6 weeks | 148 (85%) | 150 (87%) | 152 (87%) | 155 (90%) | 145 (83%) | 147 (85%) |
| 6 months | 135 (78%) | 138 (80%) | 142 (82%) | 145 (84%) | 134 (77%) | 135 (78%) |

**Table S4. Quality-adjusted life years, healthcare and societal costs, and cost-effectiveness at 6 months (complete cases, without imputation for missing data)**

|  | Virtual Reality + TAU  n=100 | | TAU only  n=103 | | Adjusted Difference between arms | | |
| --- | --- | --- | --- | --- | --- | --- | --- |
|  | Mean | SE | Mean | SE | Mean | 95% CI | |
| QALYs - EQ-5D | 0.311 | 0.012 | 0.297 | 0.011 | 0.019 | (-0.003, | 0.042) |
| QALYs - ReQoL | 0.400 | 0.007 | 0.391 | 0.008 | 0.009 | (-0.007, | 0.026) |
|  |  |  |  |  |  |  |  |
| Cost of Mental Health Admissions (NHS) | £969 | £517 | £657 | £484 | -£240 | (-£1,097, | £617) |
| Medication Costs (NHS) | £220 | £30 | £160 | £17 | £44 | (-£20, | £107) |
| General Healthcare costs (NHS) | £1,445 | £136 | £1,508 | £150 | -£136 | (-£511, | £239) |
| Paid Help Costs (NHS/PSS) | £29 | £19 | £11 | £7 | £16 | (-£24, | £57) |
| Total NHS & PSS Costs | £2,718 | £909 | £2,661 | £845 | -£32 | (-£719, | £656) |
|  |  |  |  |  |  |  |  |
| Criminal Justice Costs | £55 | £28 | £3 | £3 | £47 | (-£12, | £105) |
| Unpaid Caregiving (societal cost) | £2,882 | £460 | £3,965 | £767 | -£1,118 | (-£2,888, | £652) |
| Total Private Healthcare | £51 | £18 | £145 | £40 | -£105 | (-£185, | -£26) |
| Total Societal Costs | £5,642 | £1,065 | £6,892 | £1,198 | -£1,516 | (-£3,661, | £630) |
|  |  |  |  |  |  |  |  |
| **Using EQ-5D Utilities (per NICE guidance)** | **Maximum cost-effective price**  **Threshold: £20,000 per QALY** | | | **Maximum cost-effective price**  **Threshold: £30,000 per QALY** | | | |
| Total NHS & PSS Costs (per NICE guidance) | £417 | | | £610 | | | |
| Total Societal Costs | £1,901 | | | £2,094 | | | |
| **Using ReQoL utilities** |  |  |  |  |  |  |  |
| Total NHS & PSS Costs | £222 | | | £317 | | | |
| Total Societal Costs | £1,706 | | | £1,801 | | | |
|  |  |  |  |  |  |  |  |

TAU: Treatment as usual. QALY: Quality adjusted life year.

Cost differences between treatment arms were obtained from a linear regression model, adjusted for treatment allocation, randomised service and site. QALY differences between treatment arms were obtained from a linear regression model, adjusted for treatment allocation, baseline utility, randomised service and site.

The maximum cost-effective price of the gameChange intervention is estimated at the lower and upper bound of UK willingness to pay for health interventions, £20,000 per QALY or £30,000 per QALY respectively. This represents the maximum price which can be charged for a patient’s gameChange treatment that remains cost-effective, at the lower and upper bound of the cost-effectiveness threshold.

**Figure S1. Cost-effectiveness planes using EQ5D and ReQoL QALYs for each costing perspective (complete cases, without imputation for missing data)**


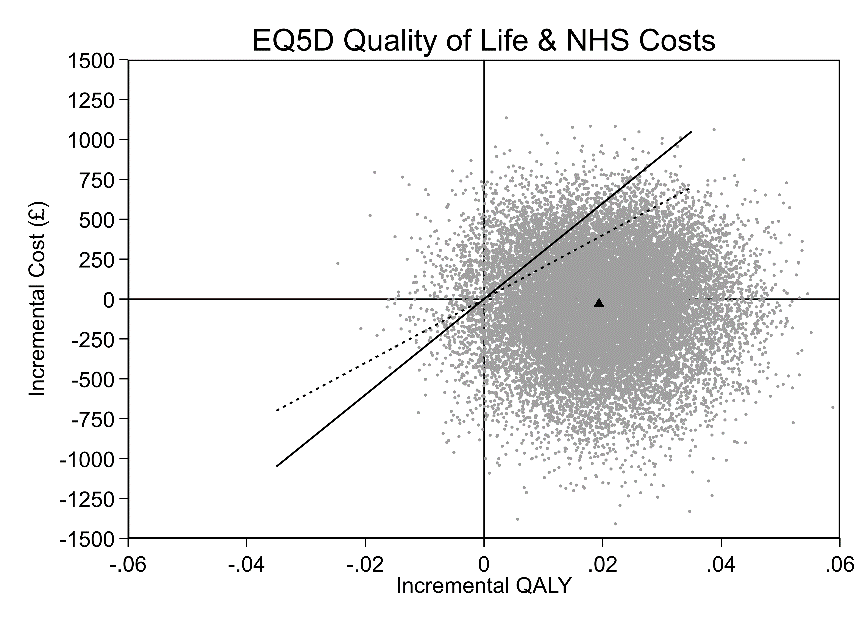

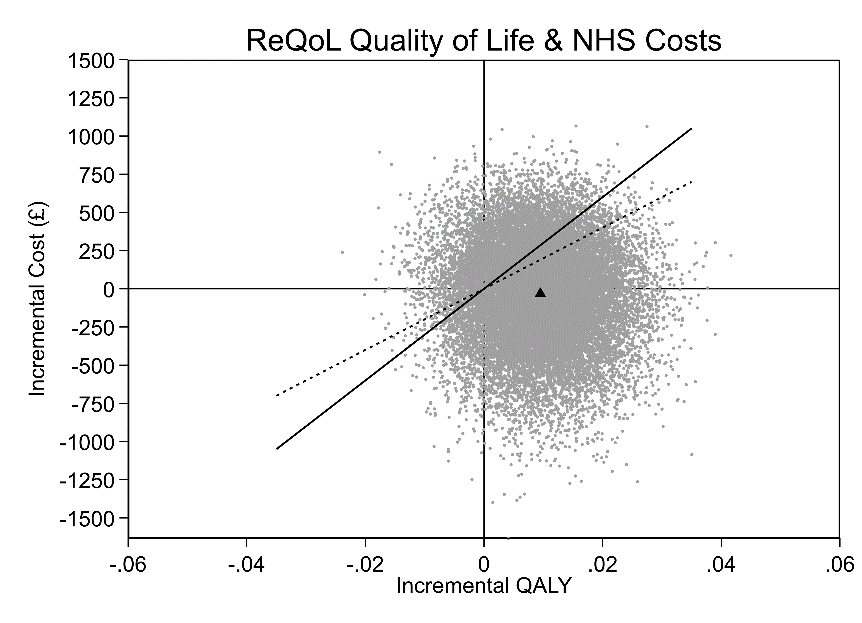

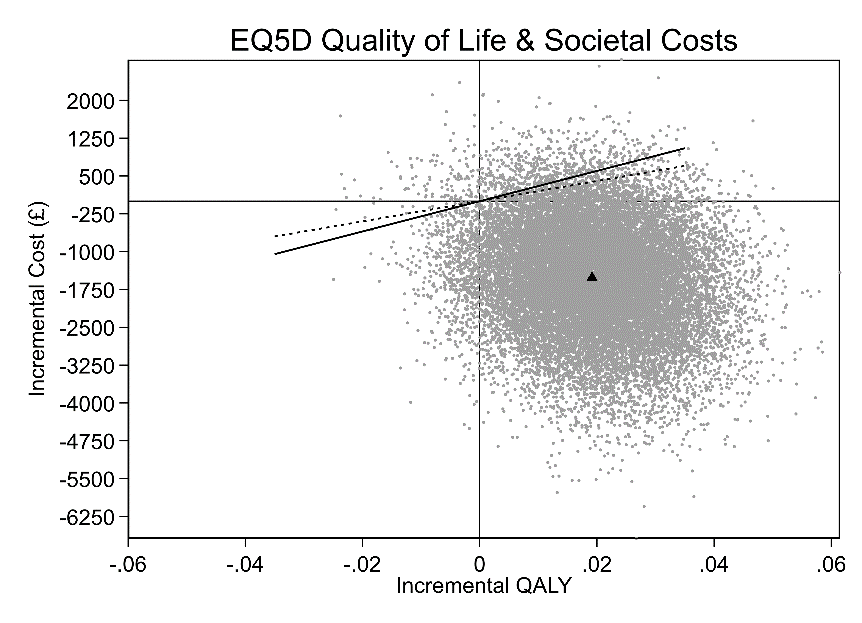

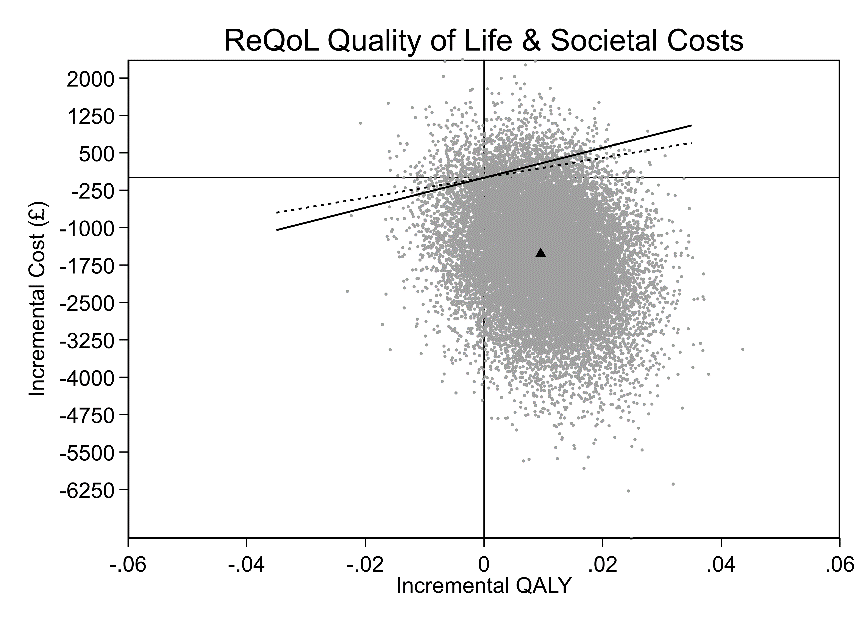


Cost saving & Health improving

Cost saving & Health worsening

Cost increasing & Health improving

Cost increasing & Health worsening

Cost saving & Health improving

Cost saving & Health worsening

Cost increasing & Health improving

Cost increasing & Health worsening

Cost saving & Health improving

Cost saving & Health worsening

Cost increasing & Health improving

Cost increasing & Health worsening

Cost saving & Health improving

Cost saving & Health worsening

Cost increasing & Health improving

Cost increasing & Health worsening

Dots represent 22,000 individual simulated pairs of mean incremental costs and effects. The triangle represents the mean incremental costs and effects across all 22,000 simulations. Straight lines intersecting the y-axis represent the UK cost-effectiveness threshold (λ), which lies between λ = £20,000 per QALY (dotted line) & λ = £30,000 per QALY (solid line).

**Figure S2. Uncertainty surrounding the maximum cost-effective price of the gameChange intervention, (complete cases, without imputation for missing data)**


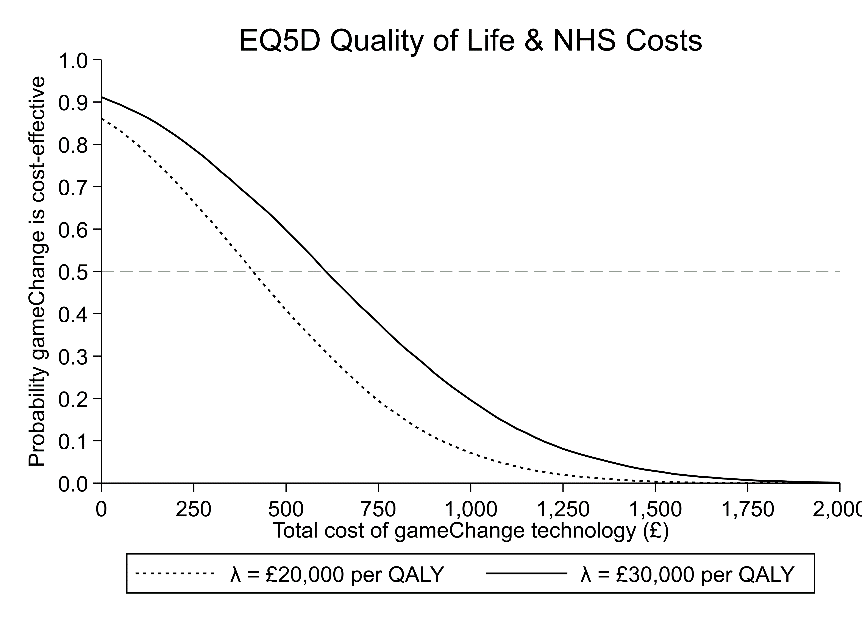

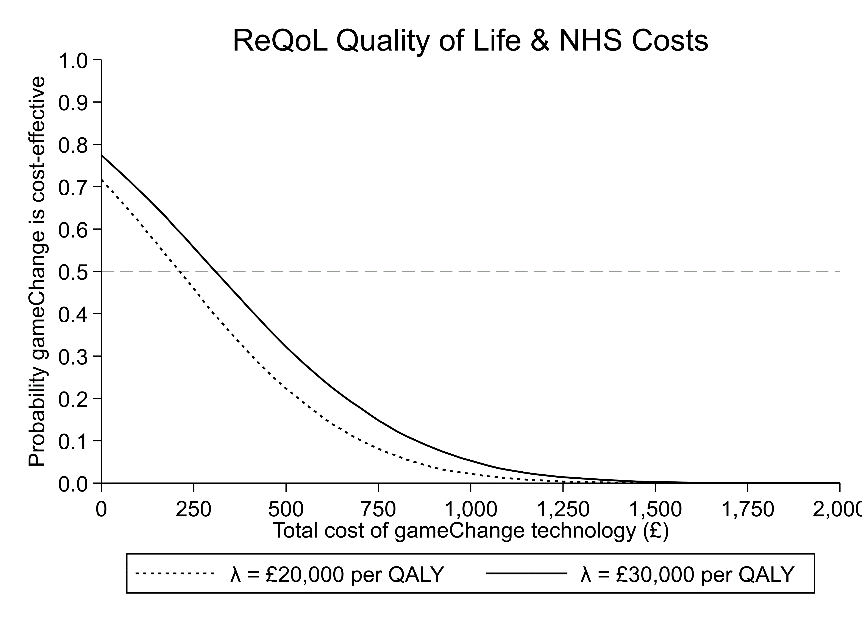

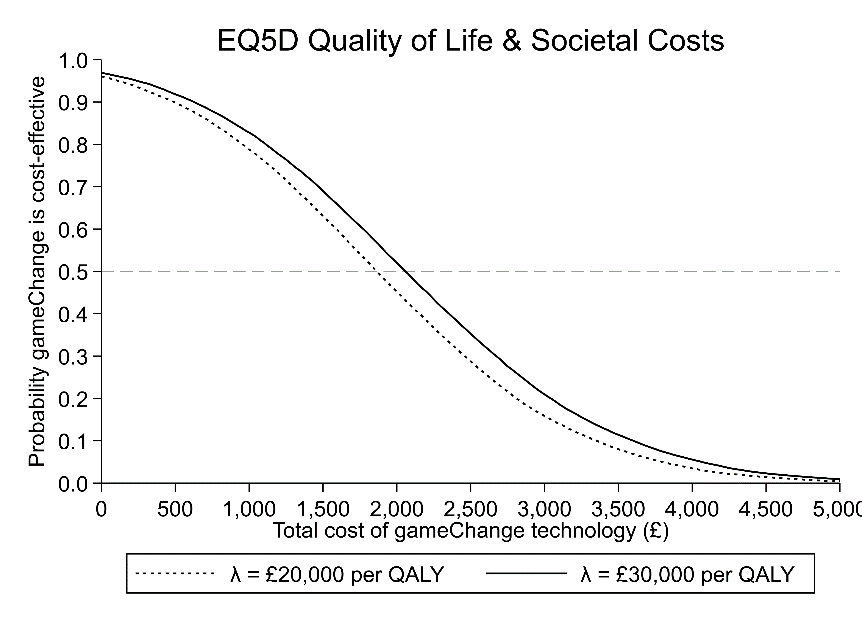

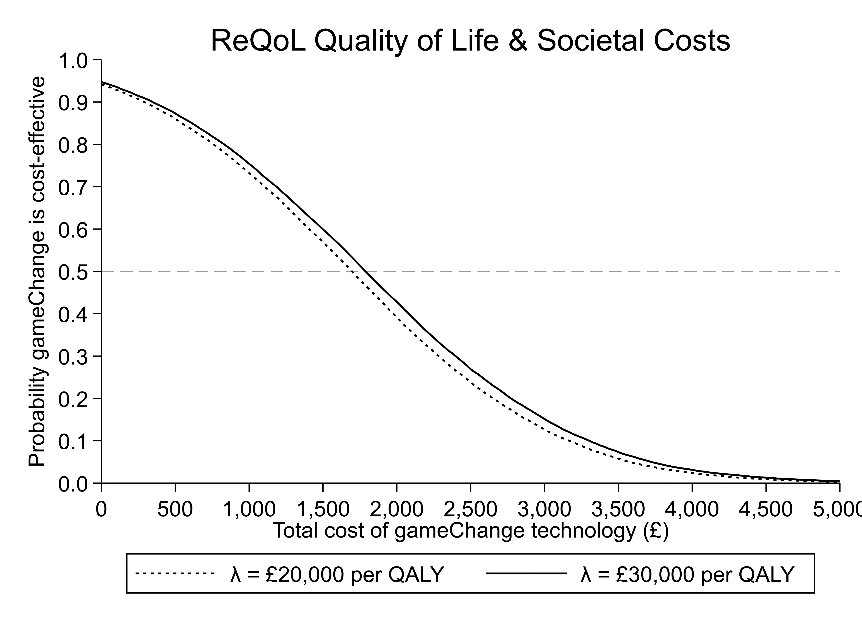


Lines represent the maximum cost-effective price of gameChange for the UK NHS at the NICE cost-effectiveness threshold (between £20,000 & £30,000 per QALY). The dotted line represents the maximum cost-effective price of the gameChange intervention at the lower bound of the cost-effectiveness threshold (λ = £20,000 per QALY), whilst the solid line represents the maximum cost-effective price of the gameChange intervention at the upper bound of the cost-effectiveness threshold (λ = £30,000 per QALY).

**Table S5. Maximum cost-effective price for gameChange in subgroups stratified by OAS Avoidance and Distress Scores (complete cases, without imputation for missing data)**

| **EQ5D Quality of Life Measure** | | | | | **Maximum cost-effective price** | |
| --- | --- | --- | --- | --- | --- | --- |
| **NHS/PSS Perspective** | *n (VR+TAU)* | *n (TAU)* | *Incremental QALY (95% CI)* | *Incremental Cost (95% CI)* | *λ= £20,000 per QALY* | *λ= £30,000 per QALY* |
| **Overall Sample** | 100 | 103 | 0.019 (-0.003, 0.042) | -£32 (-£719, £656) | £417 | £610 |
| High or Severe Avoidance | 43 | 56 | 0.023 (-0.013, 0.059) | -£36 (-£653, £581) | £496 | £726 |
| High or Severe Distress | 55 | 66 | 0.023 (-0.010, 0.057) | £5 (-£636, £645) | £463 | £697 |
| High or Severe Avoidance or Distress | 64 | 69 | 0.025 (-0.006, 0.056) | -£13 (-£600, £574) | £512 | £761 |
| High or Severe Avoidance and Distress | 34 | 53 | 0.021 (-0.021, 0.062) | £10 (-£695, £715) | £402 | £608 |
| **Societal Perspective** | *n (VR+TAU)* | *n (TAU)* | *Incremental QALY (95% CI)* | *Incremental Cost (95% CI)* | *λ= £20,000 per QALY* | *λ= £30,000 per QALY* |
| **Overall Sample** | 100 | 103 | 0.019 (-0.003, 0.042) | -£1516 (-£3661, £630) | £1,901 | £2,094 |
| High or Severe Avoidance | 43 | 56 | 0.023 (-0.013, 0.059) | -£1303 (-£5187, £2582) | £1,763 | £1,993 |
| High or Severe Distress | 55 | 66 | 0.023 (-0.010, 0.057) | -£1187 (-£4446, £2073) | £1,655 | £1,889 |
| High or Severe Avoidance or Distress | 64 | 69 | 0.025 (-0.006, 0.056) | -£1355 (-£4349, £1639) | £1,854 | £2,103 |
| High or Severe Avoidance and Distress | 34 | 53 | 0.021 (-0.021, 0.062) | -£831 (-£5244, £3582) | £1,243 | £1,449 |
| **ReQoL Quality of Life Measure** | | | | | **Maximum cost-effective price** | |
| **NHS/PSS Perspective** | *n (VR+TAU)* | *n (TAU)* | *Incremental QALY (95% CI)* | *Incremental Cost (95% CI)* | *λ= £20,000 per QALY* | *λ= £30,000 per QALY* |
| **Overall Sample** | 100 | 103 | 0.009 (-0.007, 0.026) | -£32 (-£719, £656) | £222 | £317 |
| High or Severe Avoidance | 43 | 56 | 0.023 (-0.005, 0.050) | -£36 (-£653, £581) | £488 | £715 |
| High or Severe Distress | 55 | 66 | 0.014 (-0.010, 0.037) | £5 (-£636, £645) | £269 | £406 |
| High or Severe Avoidance or Distress | 64 | 69 | 0.016 (-0.006, 0.038) | -£13 (-£600, £574) | £336 | £498 |
| High or Severe Avoidance and Distress | 34 | 53 | 0.020 (-0.011, 0.050) | £10 (-£695, £715) | £384 | £582 |
| **Societal Perspective** | *n (VR+TAU)* | *n (TAU)* | *Incremental QALY (95% CI)* | *Incremental Cost (95% CI)* | *λ= £20,000 per QALY* | *λ= £30,000 per QALY* |
| **Overall Sample** | 100 | 103 | 0.009 (-0.007, 0.026) | -£1516 (-£3661, £630) | £1,706 | £1,801 |
| High or Severe Avoidance | 43 | 56 | 0.023 (-0.005, 0.050) | -£1303 (-£5187, £2582) | £1,755 | £1,981 |
| High or Severe Distress | 55 | 66 | 0.014 (-0.010, 0.037) | -£1187 (-£4446, £2073) | £1,460 | £1,597 |
| High or Severe Avoidance or Distress | 64 | 69 | 0.016 (-0.006, 0.038) | -£1355 (-£4349, £1639) | £1,678 | £1,840 |
| High or Severe Avoidance and Distress | 34 | 53 | 0.020 (-0.011, 0.050) | -£831 (-£5244, £3582) | £1,225 | £1,422 |

VR + TAU: Virtual Reality therapy in addition to treatment as usual. TAU: Treatment as usual.

The maximum cost-effective price for the gameChange intervention was estimated at the lower (£20,000 per QALY) and upper (£30,000 per QALY) bound of the UK cost-effectiveness threshold (λ), representing the willingness to pay for health interventions. The maximum cost-effective price therefore represents the maximum price which can be charged for a patient’s virtual reality therapy, that remains cost-effective at the lower and upper bound of the cost-effectiveness threshold.

1. Available data

**Table S6: Resource use counts. Mean number of healthcare visits by type, treatment allocation, and follow-up period (available data)**

|  | **Baseline to 6 Weeks** | | | **6 weeks to 6 months** | | |
| --- | --- | --- | --- | --- | --- | --- |
|  | VR + TAU  Mean (SD) | TAU  Mean (SD) | Difference  (95% CI) | VR + TAU  Mean (SD) | TAU  Mean (SD) | Difference  (95% CI) |
| **NHS / Social Care** |  |  |  |  |  |  |
| GP visits | 0.8 (1.3) | 1.1 (1.9) | -0.2 (-0.7, 0.2) | 1.3 (2.3) | 1.5 (2.3) | -0.2 (-0.7, 0.2) |
| Psychiatrist visits | 0.5 (1.4) | 0.6 (1.2) | -0.1 (-0.5, 0.3) | 0.8 (1.3) | 1.1 (2.5) | -0.4 (-0.8, 0.0) |
| Therapist visits | 0.4 (1.6) | 0.3 (1.0) | 0.0 (-0.6, 0.7) | 1.3 (3.4) | 1.8 (4.4) | -0.5 (-1.2, 0.2) |
| Community Mental Health | 4.1 (7.4) | 3.5 (3.7) | 0.1 (-1.8, 2.1) | 8.2 (14.6) | 5.1 (5.7) | 2.9 (0.8, 5.0) |
| A&E visits | 0.0 (0.2) | 0.1 (0.5) | -0.1 (-0.2, 0.0) | 0.1 (0.3) | 0.2 (0.8) | -0.1 (-0.2, 0.0) |
| Outpatient care | 0.2 (0.6) | 0.3 (0.7) | -0.1 (-0.2, 0.1) | 0.2 (0.8) | 0.3 (1.0) | -0.1 (-0.3, 0.1) |
| Paid help at home | 0.9 (6.0) | 0.1 (0.5) | 0.6 (-0.7, 1.8) | 0.5 (4.4) | 1.1 (7.7) | -0.5 (-1.9, 0.8) |
| **Private** |  |  |  |  |  |  |
| GP visits | 0.0 (0.2) | 0.0 (0.2) | 0.0 (-0.1, 0.1) | 0.1 (0.4) | 0.1 (0.4) | 0.0 (-0.1, 0.1) |
| Psychiatrist visits | 0.0 (0.0) | 0.1 (0.3) | -0.1 (-0.1, 0.0) | 0.0 (0.2) | 0.1 (0.3) | 0.0 (-0.1, 0.0) |
| Therapist visits | 0.0 (0.0) | 0.0 (0.4) | 0.0 (-0.1, 0.1) | 0.1 (0.5) | 0.1 (0.7) | 0.0 (-0.1, 0.1) |
| Community Mental Health | 0.1 (0.4) | 0.0 (0.2) | 0.0 (0.0, 0.1) | 0.1 (0.4) | 0.1 (0.4) | 0.0 (-0.1, 0.1) |
| A&E visits | 0.0 (0.0) | 0.0 (0.1) | 0.0 (0.0, 0.0) | 0.0 (0.1) | 0.0 (0.2) | 0.0 (0.0, 0.0) |
| Outpatient care | 0.0 (0.0) | 0.0 (0.1) | 0.0 (0.0, 0.0) | 0.0 (0.2) | 0.0 (0.2) | 0.0 (-0.1, 0.0) |
| Paid help at home | 0.5 (4.6) | 0.4 (3.4) | -0.2 (-2.0, 1.7) | 0.5 (5.4) | 2.3 (14.5) | -2.0 (-3.9, -0.1) |

VR + TAU: Virtual Reality therapy in addition to treatment as usual. TAU: Treatment as usual.

Differences between treatment arms were obtained from multilevel mixed-effects models, adjusted for treatment allocation, randomised service and site; a time by treatment interaction was included in the model; the follow-up time point was used as a categorical variable.

**Table S7. Data values for utilities for each follow-up period (available data)**

| **EQ-5D data** | **VR + TAU** | | **TAU** | | **Adjusted Difference** | **P value** |
| --- | --- | --- | --- | --- | --- | --- |
|  | **n=** | **Mean (SD)** | **n=** | **Mean (SD)** | **(95% CI)** |  |
| **Baseline** | 172 (99%) | 0.538 (0.274) | 170 (99%) | 0.545 (0.268) |  |  |
| **6 weeks** | 152 (87%) | 0.603 (0.277) | 155 (90%) | 0.591 (0.276) | 0.030 (-0.019, 0.078) | .231 |
| **6 months** | 142 (82%) | 0.559 (0.295) | 145 (84%) | 0.574 (0.278) | -0.004 (-0.054, 0.045) | .863 |
| **ReQoL data** | **VR + TAU** | | **TAU** | | **Adjusted Difference** | **P value** |
|  | **n=** | **Mean (SD)** | **n=** | **Mean (SD)** | **(95% CI)** |  |
| **Baseline** | 162 (93%) | 0.732 (0.223) | 158 (92%) | 0.746 (0.201) |  |  |
| **6 weeks** | 145 (83%) | 0.776 (0.204) | 147 (85%) | 0.773 (0.204) | 0.021 (-0.018, 0.059) | .292 |
| **6 months** | 134 (77%) | 0.776 (0.197) | 135 (78%) | 0.795 (0.170) | -0.013 (-0.053, 0.026) | .510 |

VR + TAU: Virtual Reality therapy in addition to treatment as usual. TAU: Treatment as usual.

Differences between treatment arms were obtained from multilevel mixed-effects models, adjusted for treatment allocation, baseline utility, randomised service and site; a time by treatment interaction was included in the model; the follow-up time point was used as a categorical variable.

**Table S8: Data values for costs for each follow-up period (available data)**

|  | **Baseline to 6 Weeks** | | | **6 weeks to 6 months** | | |
| --- | --- | --- | --- | --- | --- | --- |
|  | **VR + TAU**  **n=136** | **TAU**  **n=137** | **Adjusted**  **Difference** | **VR + TAU**  **n=125** | **TAU**  **n=123** | **Adjusted**  **Difference** |
|  | **Mean (SD)** | **Mean (SD)** | **(95%CI)** | **Mean (SD)** | **Mean (SD)** | **(95%CI)** |
| **Total NHS costs** | 869 (234) | 668 (143) | -248 (-1005, 510) | 2059 (592) | 1740 (567) | 85 (-708, 879) |
| **Mental Health Inpatient Stays*** | *265 (157)* | *105 (105)* | *-116 (-696, 463)* | *703 (388)* | *551 (384)* | *-124 (-703, 455)* |
| **Physical Health Inpatient Stays*** | *91 (91)* | *23 (23)* | *74 (-137, 285)* | *147 (120)* | *11 (9)* | *141 (-70, 352)* |
| **Medication costs*** | *51 (7)* | *37 (5)* | *7 (-31, 44)* | *168 (24)* | *123 (14)* | *37 (0, 74)* |
| **GP visits** | 27 (5) | 33 (5) | -6 (-22, 11) | 46 (9) | 43 (6) | -1 (-18, 16) |
| **Psychiatrist visits** | 180 (41) | 196 (34) | -34 (-167, 100) | 272 (39) | 394 (79) | -132 (-271, 8) |
| **Therapist visits** | 24 (9) | 21 (6) | -3 (-58, 53) | 120 (28) | 140 (33) | -20 (-78, 38) |
| **Community Mental Health** | 190 (30) | 163 (15) | 9 (-81, 99) | 376 (58) | 235 (24) | 131 (37, 224) |
| **A&E visits** | 5 (3) | 17 (8) | -14 (-34, 5) | 13 (4) | 34 (12) | -19 (-39, 1) |
| **Outpatient care** | 28 (8) | 39 (10) | -10 (-38, 18) | 35 (11) | 55 (14) | -21 (-50, 9) |
| **Paid help at home** | 21 (12) | 1 (1) | 13 (-17, 42) | 11 (9) | 25 (16) | -12 (-43, 18) |

VR + TAU: Virtual Reality therapy in addition to treatment as usual. TAU: Treatment as usual.

Differences between treatment arms were obtained from multilevel mixed-effects models, adjusted for treatment allocation, randomised service and site; a time by treatment interaction was included in the model; the follow-up time point was used as a categorical variable.

* Complete data available for Mental Health Inpatient Stays, Physical Health Inpatient Stays & Medication costs:

(VR + TAU) = 174, (TAU) = 172.

**Table S9. Response level data for EQ-5D questionnaires by follow-up time and treatment allocation (available data)**

VR + TAU: Virtual Reality therapy in addition to treatment as usual. TAU: Treatment as usual.

|  | **Baseline** | | **6 Weeks** | | **6 Months** | |
| --- | --- | --- | --- | --- | --- | --- |
| **Domain** | *VR+TAU* | *TAU* | *VR+TAU* | *TAU* | *VR+TAU* | *TAU* |
|  |  |  |  |  |  |  |
| **Mobility** (available data) | 173 (99%) | 171 (99%) | 153 (88%) | 156 (91%) | 143 (82%) | 146 (85%) |
|  |  |  |  |  |  |  |
| I have no problems walking about | 106 (61%) | 103 (60%) | 99 (65%) | 93 (60%) | 76 (53%) | 93 (64%) |
| I have slight problems walking about | 21 (12%) | 31 (18%) | 22 (14%) | 31 (20%) | 22 (15%) | 21 (14%) |
| I have moderate problems walking about | 29 (17%) | 27 (16%) | 20 (13%) | 20 (13%) | 32 (22%) | 21 (14%) |
| I have severe problems walking about | 17 (10%) | 7 (4%) | 11 (7%) | 10 (6%) | 13 (9%) | 9 (6%) |
| I am unable to walk about | 0 (0%) | 3 (2%) | 1 (1%) | 2 (1%) | 0 (0%) | 2 (1%) |
|  |  |  |  |  |  |  |
| **Self-care** (available data) | 173 (99%) | 171 (99%) | 152 (87%) | 155 (90%) | 142 (82%) | 146 (85%) |
|  |  |  |  |  |  |  |
| I have no problems walking about | 95 (55%) | 101 (59%) | 94 (62%) | 90 (58%) | 77 (54%) | 95 (65%) |
| I have slight problems walking about | 37 (21%) | 39 (23%) | 25 (16%) | 37 (24%) | 30 (21%) | 26 (18%) |
| I have moderate problems walking about | 34 (20%) | 22 (13%) | 25 (16%) | 23 (15%) | 26 (18%) | 17 (12%) |
| I have severe problems walking about | 7 (4%) | 9 (5%) | 8 (5%) | 5 (3%) | 9 (6%) | 7 (5%) |
| I am unable to walk about | 0 (0%) | 0 (0%) | 0 (0%) | 0 (0%) | 0 (0%) | 1 (1%) |
|  |  |  |  |  |  |  |
| **Usual activities** (available data) | 173 (99%) | 171 (99%) | 153 (88%) | 156 (91%) | 143 (82%) | 146 (85%) |
|  |  |  |  |  |  |  |
| I have no problems walking about | 37 (21%) | 46 (27%) | 44 (29%) | 49 (31%) | 41 (29%) | 45 (31%) |
| I have slight problems walking about | 44 (25%) | 37 (22%) | 48 (31%) | 45 (29%) | 35 (24%) | 36 (25%) |
| I have moderate problems walking about | 56 (32%) | 56 (33%) | 41 (27%) | 42 (27%) | 42 (29%) | 38 (26%) |
| I have severe problems walking about | 27 (16%) | 24 (14%) | 17 (11%) | 17 (11%) | 23 (16%) | 21 (14%) |
| I am unable to walk about | 9 (5%) | 8 (5%) | 3 (2%) | 3 (2%) | 2 (1%) | 6 (4%) |
|  |  |  |  |  |  |  |
| **Pain/Discomfort** (available data) | 172 (99%) | 170 (99%) | 152 (87%) | 156 (91%) | 143 (82%) | 146 (85%) |
|  |  |  |  |  |  |  |
| I have no problems walking about | 77 (45%) | 79 (46%) | 84 (55%) | 77 (49%) | 61 (43%) | 71 (49%) |
| I have slight problems walking about | 43 (25%) | 37 (22%) | 28 (18%) | 32 (21%) | 32 (22%) | 27 (18%) |
| I have moderate problems walking about | 32 (19%) | 33 (19%) | 22 (14%) | 30 (19%) | 28 (20%) | 31 (21%) |
| I have severe problems walking about | 16 (9%) | 18 (11%) | 14 (9%) | 12 (8%) | 15 (10%) | 15 (10%) |
| I am unable to walk about | 4 (2%) | 3 (2%) | 4 (3%) | 5 (3%) | 7 (5%) | 2 (1%) |
|  |  |  |  |  |  |  |
| **Anxiety/Depression** (available data) | 172 (99%) | 171 (99%) | 153 (88%) | 156 (91%) | 144 (83%) | 145 (84%) |
|  |  |  |  |  |  |  |
| I have no problems walking about | 8 (5%) | 6 (4%) | 14 (9%) | 18 (12%) | 14 (10%) | 16 (11%) |
| I have slight problems walking about | 37 (22%) | 36 (21%) | 34 (22%) | 33 (21%) | 32 (22%) | 26 (18%) |
| I have moderate problems walking about | 64 (37%) | 65 (38%) | 62 (41%) | 62 (40%) | 56 (39%) | 53 (37%) |
| I have severe problems walking about | 32 (19%) | 41 (24%) | 26 (17%) | 25 (16%) | 23 (16%) | 27 (19%) |
| I am unable to walk about | 31 (18%) | 23 (13%) | 17 (11%) | 18 (12%) | 19 (13%) | 23 (16%) |

**Table S10. Response level data for ReQoL questionnaire items used to calculate utilities, at each follow-up time by treatment allocation (available data)**

| **Domain** | **Baseline** | | **6 Weeks** | | **6 Months** | |
| --- | --- | --- | --- | --- | --- | --- |
|  | *VR+TAU* | *TAU* | *VR+TAU* | *TAU* | *VR+TAU* | *TAU* |
|  |  |  |  |  |  |  |
| **I felt unable to cope** |  |  |  |  |  |  |
| Available data | 166 (95%) | 162 (94%) | 149 (86%) | 149 (87%) | 138 (79%) | 137 (80%) |
|  |  |  |  |  |  |  |
| None of the time | 19 (11%) | 27 (17%) | 28 (19%) | 30 (20%) | 28 (20%) | 32 (23%) |
| Only occasionally | 43 (26%) | 38 (23%) | 37 (25%) | 46 (31%) | 35 (25%) | 34 (25%) |
| Sometimes | 52 (31%) | 50 (31%) | 51 (34%) | 43 (29%) | 42 (30%) | 46 (34%) |
| Often | 30 (18%) | 32 (20%) | 22 (15%) | 21 (14%) | 23 (17%) | 16 (12%) |
| Most or all of the time | 22 (13%) | 15 (9%) | 11 (7%) | 9 (6%) | 10 (7%) | 9 (7%) |
|  |  |  |  |  |  |  |
| **I felt happy** |  |  |  |  |  |  |
| Available data | 167 (96%) | 162 (94%) | 148 (85%) | 149 (87%) | 138 (79%) | 137 (80%) |
|  |  |  |  |  |  |  |
| None of the time | 32 (19%) | 21 (13%) | 19 (13%) | 17 (11%) | 21 (15%) | 14 (10%) |
| Only occasionally | 50 (30%) | 51 (31%) | 44 (30%) | 40 (27%) | 28 (20%) | 34 (25%) |
| Sometimes | 57 (34%) | 69 (43%) | 53 (36%) | 57 (38%) | 54 (39%) | 54 (39%) |
| Often | 22 (13%) | 16 (10%) | 27 (18%) | 26 (17%) | 27 (20%) | 22 (16%) |
| Most or all of the time | 6 (4%) | 5 (3%) | 5 (3%) | 9 (6%) | 8 (6%) | 13 (9%) |
|  |  |  |  |  |  |  |
| **I thought my life was not worth living** |  |  |  |  |  |  |
| Available data | 167 (96%) | 161 (94%) | 148 (85%) | 148 (86%) | 138 (79%) | 137 (80%) |
|  |  |  |  |  |  |  |
| None of the time | 65 (39%) | 70 (43%) | 68 (46%) | 75 (51%) | 63 (46%) | 71 (52%) |
| Only occasionally | 38 (23%) | 38 (24%) | 23 (16%) | 31 (21%) | 26 (19%) | 23 (17%) |
| Sometimes | 32 (19%) | 29 (18%) | 32 (22%) | 17 (11%) | 31 (22%) | 27 (20%) |
| Often | 22 (13%) | 18 (11%) | 15 (10%) | 18 (12%) | 12 (9%) | 11 (8%) |
| Most or all of the time | 10 (6%) | 6 (4%) | 10 (7%) | 7 (5%) | 6 (4%) | 5 (4%) |
|  |  |  |  |  |  |  |
| **I enjoyed what I did** |  |  |  |  |  |  |
| **A**vailable data | 168 (97%) | 163 (95%) | 148 (85%) | 149 (87%) | 138 (79%) | 137 (80%) |
|  |  |  |  |  |  |  |
| None of the time | 19 (11%) | 14 (9%) | 13 (9%) | 15 (10%) | 19 (14%) | 11 (8%) |
| Only occasionally | 47 (28%) | 41 (25%) | 42 (28%) | 31 (21%) | 33 (24%) | 32 (23%) |
| Sometimes | 70 (42%) | 68 (42%) | 58 (39%) | 59 (40%) | 45 (33%) | 52 (38%) |
| Often | 27 (16%) | 29 (18%) | 24 (16%) | 29 (19%) | 31 (22%) | 30 (22%) |
| Most or all of the time | 5 (3%) | 11 (7%) | 11 (7%) | 15 (10%) | 10 (7%) | 12 (9%) |

| **Domain** | **Baseline** | | **6 Weeks** | | **6 Months** | |
| --- | --- | --- | --- | --- | --- | --- |
|  | *VR+TAU* | *TAU* | *VR+TAU* | *TAU* | *VR+TAU* | *TAU* |
|  |  |  |  |  |  |  |
| **I felt lonely** |  |  |  |  |  |  |
| Available data | 168 (97%) | 163 (95%) | 149 (86%) | 147 (85%) | 138 (79%) | 137 (80%) |
|  |  |  |  |  |  |  |
| None of the time | 23 (14%) | 23 (14%) | 18 (12%) | 22 (15%) | 24 (17%) | 24 (18%) |
| Only occasionally | 32 (19%) | 25 (15%) | 39 (26%) | 29 (20%) | 27 (20%) | 29 (21%) |
| Sometimes | 34 (20%) | 38 (23%) | 43 (29%) | 47 (32%) | 38 (28%) | 33 (24%) |
| Often | 46 (27%) | 41 (25%) | 31 (21%) | 29 (20%) | 37 (27%) | 29 (21%) |
| Most or all of the time | 33 (20%) | 36 (22%) | 18 (12%) | 20 (14%) | 12 (9%) | 22 (16%) |
|  |  |  |  |  |  |  |
| **I felt confident in myself** |  |  |  |  |  |  |
| Available data | 168 (97%) | 162 (94%) | 148 (85%) | 149 (87%) | 137 (79%) | 136 (79%) |
|  |  |  |  |  |  |  |
| None of the time | 55 (33%) | 44 (27%) | 30 (20%) | 37 (25%) | 31 (23%) | 29 (21%) |
| Only occasionally | 55 (33%) | 58 (36%) | 46 (31%) | 52 (35%) | 46 (34%) | 46 (34%) |
| Sometimes | 45 (27%) | 47 (29%) | 55 (37%) | 40 (27%) | 34 (25%) | 40 (29%) |
| Often | 9 (5%) | 7 (4%) | 14 (9%) | 13 (9%) | 18 (13%) | 13 (10%) |
| Most or all of the time | 4 (2%) | 6 (4%) | 3 (2%) | 7 (5%) | 8 (6%) | 8 (6%) |
|  |  |  |  |  |  |  |
| **Please describe your physical health (problems with pain, mobility, difficulties caring for yourself or feeling physically unwell) over the last week** | | | | | | |
| Available data | 166 (95%) | 163 (95%) | 148 (85%) | 149 (87%) | 135 (78%) | 136 (79%) |
|  |  |  |  |  |  |  |
| No problems | 50 (30%) | 49 (30%) | 55 (37%) | 49 (33%) | 43 (32%) | 51 (38%) |
| Slight problems | 48 (29%) | 46 (28%) | 44 (30%) | 39 (26%) | 41 (30%) | 36 (26%) |
| Moderate problems | 45 (27%) | 43 (26%) | 33 (22%) | 42 (28%) | 34 (25%) | 36 (26%) |
| Severe problems | 20 (12%) | 25 (15%) | 14 (9%) | 17 (11%) | 12 (9%) | 12 (9%) |
| Very severe problems | 3 (2%) | 0 (0%) | 2 (1%) | 2 (1%) | 5 (4%) | 1 (1%) |

VR + TAU: Virtual Reality therapy in addition to treatment as usual. TAU: Treatment as usual.

1. Multiply imputed analysis

**Figure S3: Cost-effectiveness planes using EQ5D and ReQoL QALYs for each costing perspective, following multiple imputation**


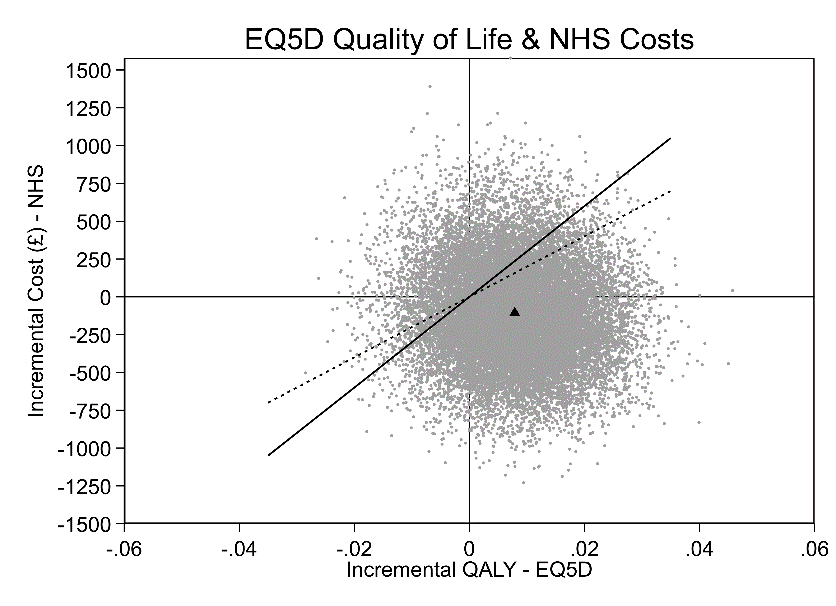

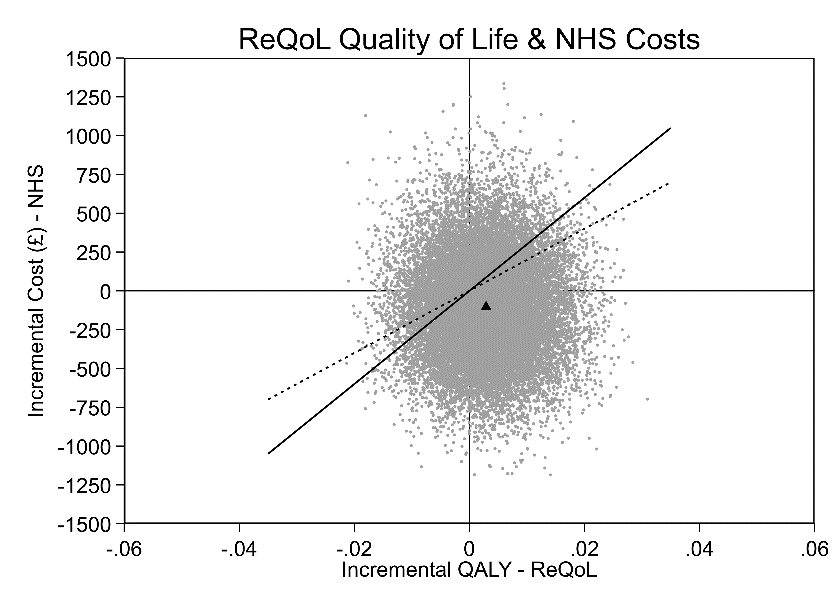

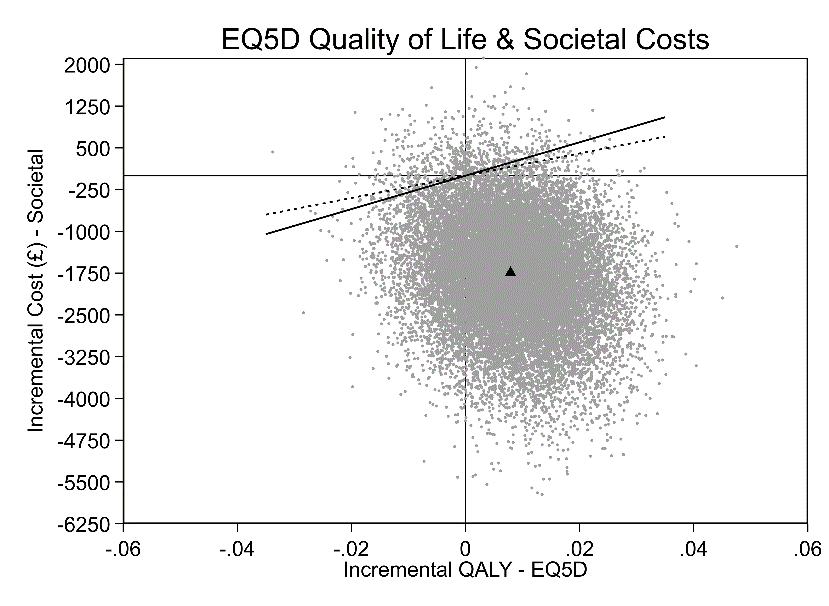

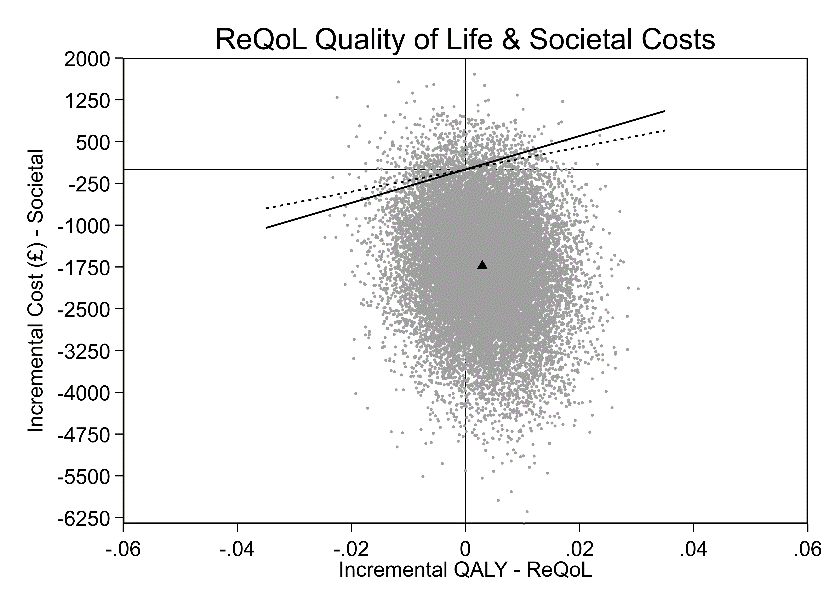


Cost saving & Health improving

Cost increasing & Health worsening

Cost increasing & Health improving

Cost saving & Health worsening

Cost saving & Health improving

Cost increasing & Health worsening

Cost increasing & Health improving

Cost saving & Health worsening

Cost saving & Health improving

Cost increasing & Health worsening

Cost increasing & Health improving

Cost saving & Health worsening

Cost saving & Health worsening

Cost saving & Health improving

Cost increasing & Health worsening

Dots represent 22,000 individual simulated pairs of mean incremental costs and effects. The triangle represents the mean incremental costs and effects across all 22,000 simulations. Straight lines intersecting the y-axis represent the UK cost-effectiveness threshold (λ), which lies between λ = £20,000 per QALY (dotted line) & λ = £30,000 per QALY (solid line).

Cost increasing & Health improving

**Figure S4: Maximum cost-effective price for the gameChange intervention in a subgroup of patients with High or Severe OAS Avoidance scores, following multiple imputation**

**
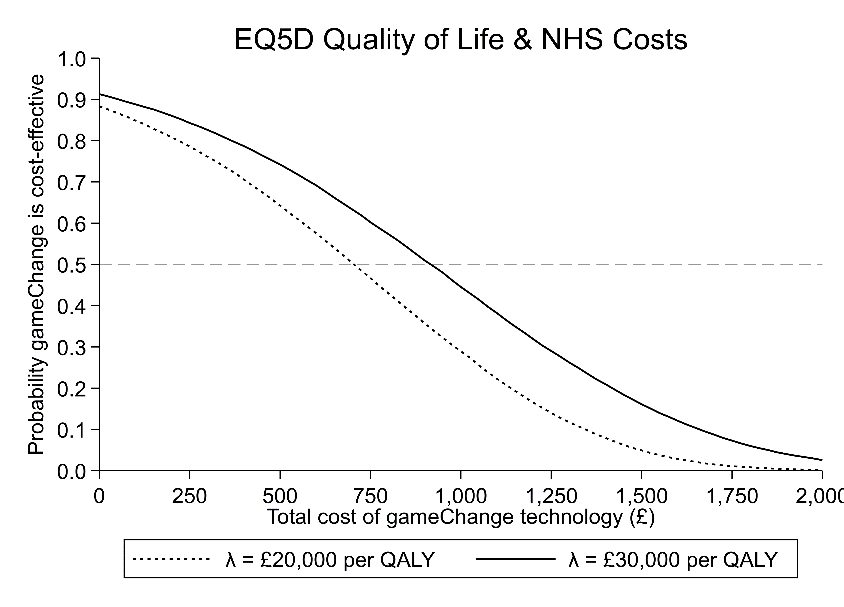

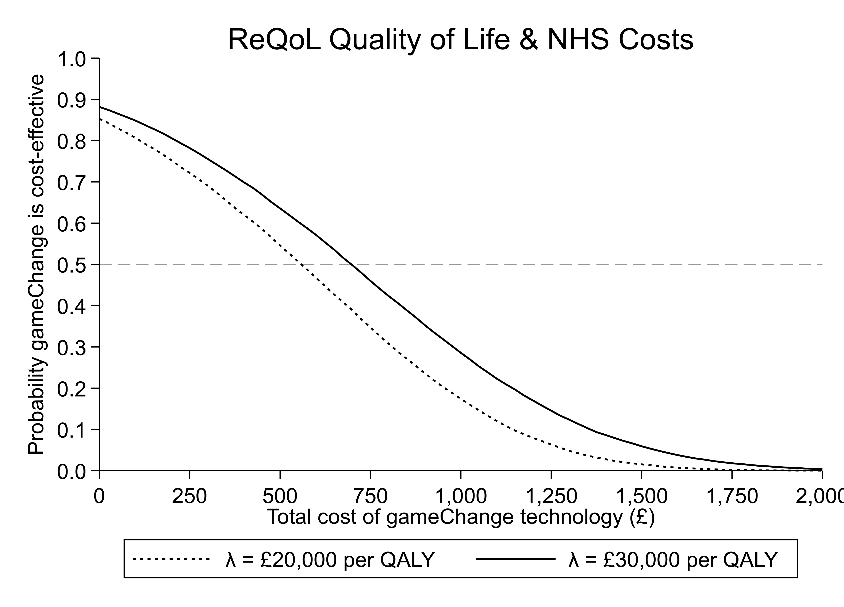

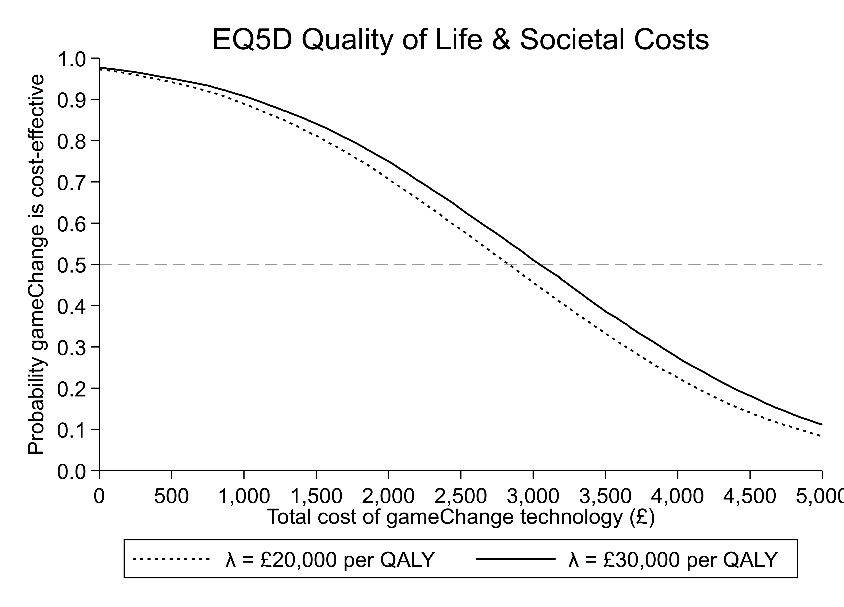

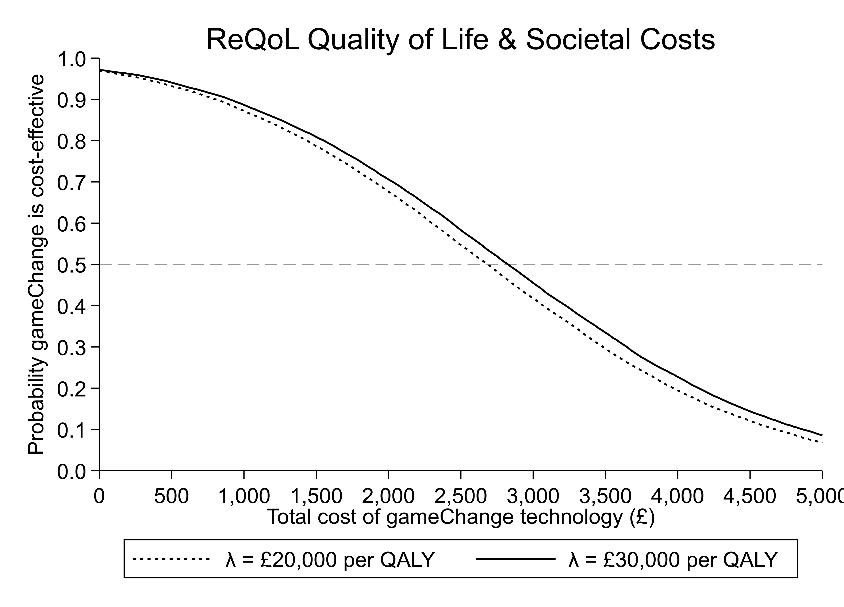
**

Lines represent the maximum cost-effective price of gameChange for the UK NHS at the NICE cost-effectiveness threshold (between £20,000 & £30,000 per QALY). The dotted line represents the maximum cost-effective price of the gameChange intervention at the lower bound of the cost-effectiveness threshold (λ = £20,000 per QALY), whilst the solid line represents the maximum cost-effective price of the gameChange intervention at the upper bound of the cost-effectiveness threshold (λ = £30,000 per QALY).

1. Sensitivity analysis 1 & 2

**Table S11: Sensitivity Analysis 1:Costs by category for patients randomised from psychiatric inpatient services at baseline, following multiple imputation**

|  | **Baseline to 6 Weeks** | | | **6 weeks to 6 months** | | |
| --- | --- | --- | --- | --- | --- | --- |
|  | **VR + TAU**  **n=3** | **TAU**  **n=1** | **P value*** | **VR + TAU**  **n=3** | **TAU**  **n=1** | **P value*** |
|  | **Mean (SE)** | **Mean (SE)** |  | **Mean (SE)** | **Mean (SE)** |  |
| **Total NHS & PSS costs** | £14,569 (£7,078) | £18,529 (-) | .820 | £31,021 (£18,516) | £65,994 (-) | .496 |
| **Mental Health Inpatient Stays** | £12,068 (£6,034) | £18,102 (-) | .693 | £26,291 (£18,533) | £62,064 (-) | .489 |
| **Physical Health Inpatient Stays** | £0 (-) | £0 (-) | - | £0 (-) | £0 (-) | - |
| **Medication costs** | £250 (£156) | £103 (-) | .708 | £1,182 (£390) | £346 (-) | .453 |
| **GP visits** | £231 (£154) | 180 (-) | .892 | £269 (£244) | £180 (-) | .878 |
| **Psychiatrist visits** | £1,605 (£1,277) | £0 (-) | .628 | £287 (-) | £3,096 (-) | - |
| **Therapist visits** | £294 (£294) | £0 (-) | .693 | £639 (£615) | £116 (-) | .734 |
| **Community Mental Health** | £120 (£120) | £144 (-) | .934 | £2,281 (£1,740) | £192 (-) | .631 |
| **A&E visits** | £0 (-) | £0 (-) | - | £0 (-) | £0 (-) | - |
| **Outpatient care** | £0 (-) | £0 (-) | - | £39 (-) | £0 (-) | - |
| **Paid help at home** | £0 (-) | £0 (-) | - | £33 (-) | £0 (-) | - |

VR + TAU: Virtual Reality therapy in addition to treatment as usual. TAU: Treatment as usual.

* p-value of difference between arms estimated via linear regression adjusted only for treatment allocation. No adjustment was made for any other covariates given small patient numbers.

**Table S12: Sensitivity Analysis 1:Utilities by category for patients randomised from psychiatric inpatient services at baseline, following multiple imputation**

| **EQ-5D data** | **VR + TAU**  **n=3**  **Mean (SE)** | **TAU**  **n=1**  **Mean (SE)** | **P value*** |
| --- | --- | --- | --- |
| **Baseline** | 0.636 (0.043) | 0.606 (-) | - |
| **6 weeks** | 0.788 (0.042) | 0.328 (-) | .085 |
| **6 months** | 0.679 (0.063) | 0.819 (-) | .441 |
| **ReQoL data** | ***VR + TAU***  ***n=3***  ***Mean (SE)*** | ***TAU***  ***n=1***  ***Mean (SE)*** | ***P value**** |
| **Baseline** | 0.735 (0.053) | 0.842 (-) | - |
| **6 weeks** | 0.714 (0.171) | 0.909 (-) | .625 |
| **6 months** | 0.751 (0.158) | 0.865 (-) | .737 |

VR + TAU: Virtual Reality therapy in addition to treatment as usual. TAU: Treatment as usual.

* p-value of difference between arms estimated via linear regression adjusted only for treatment allocation. No adjustment was made for any other covariates given small patient numbers.

Table S13: Sensitivity Analysis 1: Maximum cost-effective price for gameChange in subgroups stratified by OAS Avoidance and Distress Scores excluding psychiatric inpatients at baseline, following multiple imputation

| **EQ5D Quality of Life Measure** | | | | | **Maximum cost-effective price** | |
| --- | --- | --- | --- | --- | --- | --- |
| **NHS/PSS Perspective** | *n (VR+TAU)* | *n (TAU)* | *Incremental QALY (95% CI)* | *Incremental Cost (95% CI)* | *λ= £20,000 per QALY* | *λ= £30,000 per QALY* |
| **Overall Sample (including inpatients)** | *174* | *172* | *0.008 (-0.010, 0.026)* | *-£105 (-£1135, £924)* | *£262* | *£341* |
| **Overall Sample (excluding inpatients)** | 171 | 171 | 0.006 (-0.012, 0.025) | £233 (-£417, £883) | N/A | N/A |
| High or Severe Avoidance | 88 | 98 | 0.020 (-0.005, 0.045) | £274 (-£699, £1248) | £125 | £324 |
| High or Severe Distress | 103 | 116 | 0.008 (-0.017, 0.034) | £268 (-£576, £1112) | N/A | N/A |
| High or Severe Avoidance or Distress | 121 | 125 | 0.013 (-0.010, 0.036) | £290 (-£484, £1065) | N/A | £93 |
| High or Severe Avoidance and Distress | 70 | 89 | 0.015 (-0.014, 0.044) | 267 (-£838, £1371) | £31 | £180 |
| **Societal Perspective** | *n (VR+TAU)* | *n (TAU)* | *Incremental QALY (95% CI)* | *Incremental Cost (95% CI)* | *λ= £20,000 per QALY* | *λ= £30,000 per QALY* |
| **Overall Sample (including inpatients)** | *174* | *172* | *0.008 (-0.010, 0.026)* | *-£1731 (-£3886, £424)* | *£1,888* | *£1,967* |
| **Overall Sample (excluding inpatients)** | 171 | 171 | 0.006 (-0.012, 0.025) | -£1315 (-£3314, £683) | £1,435 | £1,495 |
| High or Severe Avoidance | 88 | 98 | 0.020 (-0.005, 0.045) | -£1911 (-£5195, £1374) | £2,310 | £2,509 |
| High or Severe Distress | 103 | 116 | 0.008 (-0.017, 0.034) | -£1583 (-£4470, £1305) | £1,749 | £1,832 |
| High or Severe Avoidance or Distress | 121 | 125 | 0.013 (-0.010, 0.036) | -£1667 (-£4310, £976) | £1,923 | £2,051 |
| High or Severe Avoidance and Distress | 70 | 89 | 0.015 (-0.014, 0.044) | -£1704 (-£5446, £2038) | £2,002 | £2,151 |

**Figure S5: Sensitivity Analysis 1:** **Maximum cost-effective price for gameChange in patients with High or Severe OAS Avoidance scores (excluding psychiatric inpatients at baseline), following multiple imputation**

**
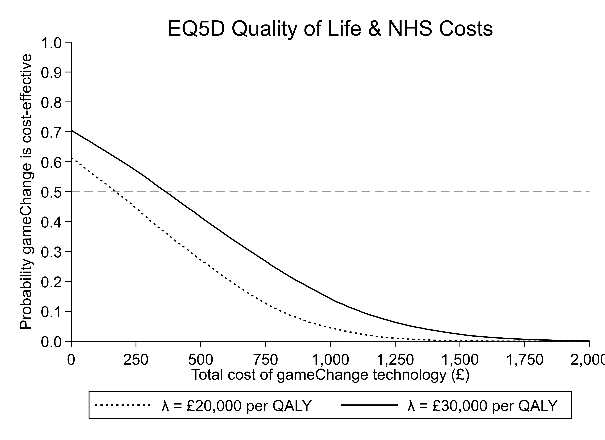

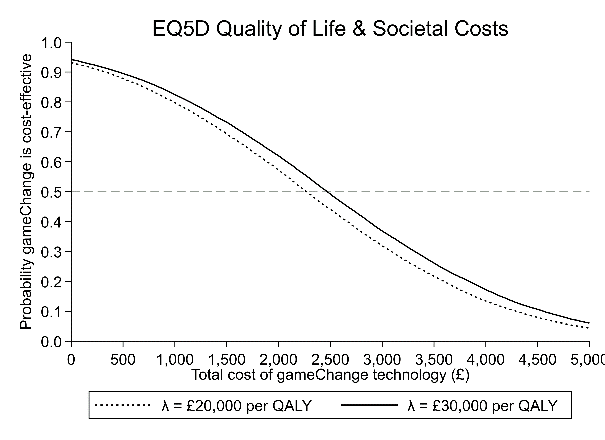
**

VR + TAU: Virtual Reality therapy in addition to treatment as usual. TAU: Treatment as usual.

The maximum cost-effective price of the gameChange intervention was estimated at the lower (£20,000 per QALY) and upper (£30,000 per QALY) bound of the UK cost-effectiveness threshold (λ), representing the willingness to pay for health interventions. The maximum cost-effective price therefore represents the maximum price which can be charged for a patient’s virtual reality therapy, that remains cost-effective at the lower and upper bound of the cost-effectiveness threshold.

**Table S14: Sensitivity Analysis 2: Maximum cost-effective price for gameChange in subgroups stratified by OAS Avoidance and Distress Scores using the Hernández Alava et al [26] EQ-5D mapping approach, following multiple imputation**

| **EQ5D Quality of Life Measure** | | | | | **Maximum cost-effective price** | |
| --- | --- | --- | --- | --- | --- | --- |
| **NHS/PSS Perspective** | *n (VR+TAU)* | *n (TAU)* | *Incremental QALY (95% CI)* | *Incremental Cost (95% CI)* | *λ= £20,000 per QALY* | *λ= £30,000 per QALY* |
| **Overall Sample** | 174 | 172 | 0.007 (-0.011, 0.026) | -£114 (-£1147, £918) | £261 | £335 |
| High or Severe Avoidance | 90 | 99 | 0.018 (-0.007, 0.044) | -£248 (-£2004, £1508) | £615 | £798 |
| High or Severe Distress | 106 | 117 | 0.007 (-0.018, 0.031) | -£189 (-£1697, £1318) | £325 | £393 |
| High or Severe Avoidance or Distress | 124 | 126 | 0.012 (-0.010, 0.034) | -£115 (-£1473, £1242) | £354 | £473 |
| High or Severe Avoidance and Distress | 72 | 90 | 0.012 (-0.016, 0.040) | -£369 (-£2406, £1668) | £614 | £736 |
| **Societal Perspective** | *n (VR+TAU)* | *n (TAU)* | *Incremental QALY (95% CI)* | *Incremental Cost (95% CI)* | *λ= £20,000 per QALY* | *λ= £30,000 per QALY* |
| **Overall Sample** | 174 | 172 | 0.007 (-0.011, 0.026) | -£1802 (-£3941, £338) | £1,949 | £2,023 |
| High or Severe Avoidance | 90 | 99 | 0.018 (-0.007, 0.044) | -£2490 (-£6057, £1078) | £2,856 | £3,040 |
| High or Severe Distress | 106 | 117 | 0.007 (-0.018, 0.031) | -£2256 (-£5394, £881) | £2,392 | £2,460 |
| High or Severe Avoidance or Distress | 124 | 126 | 0.012 (-0.010, 0.034) | -£2240 (-£5076, £597) | £2,478 | £2,598 |
| High or Severe Avoidance and Distress | 72 | 90 | 0.012 (-0.016, 0.040) | -£2440 (-£6533, £1654) | £2,685 | £2,807 |

**Figure S6: Sensitivity Analysis 2: Maximum cost-effective price for gameChange in patients with High or Severe OAS Avoidance scores using the Hernández Alava et al [26] EQ-5D mapping approach, following multiple imputation**

**
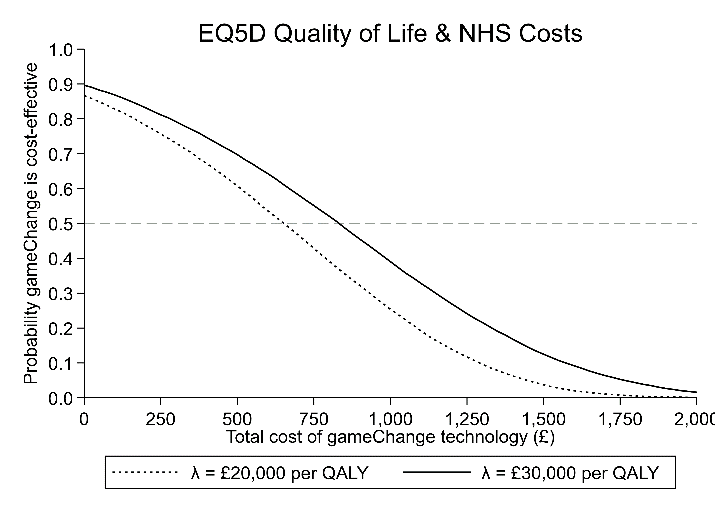

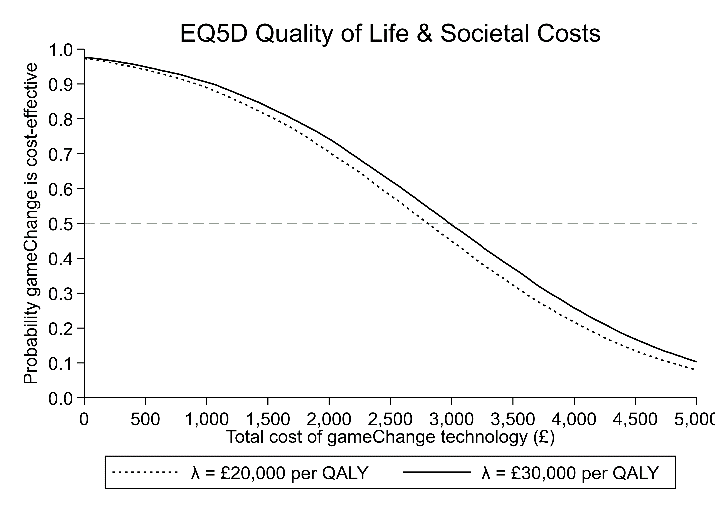
**

VR + TAU: Virtual Reality therapy in addition to treatment as usual. TAU: Treatment as usual.

The maximum cost-effective price of the gameChange intervention was estimated at the lower (£20,000 per QALY) and upper (£30,000 per QALY) bound of the UK cost-effectiveness threshold (λ), representing the willingness to pay for health interventions. The maximum cost-effective price therefore represents the maximum price which can be charged for a patient’s virtual reality therapy, that remains cost-effective at the lower and upper bound of the cost-effectiveness threshold.
